# Taxonomy-free approach overcomes the gaps in ecological knowledge: The case of foraminiferal metabarcoding applied to environmental impact assessment

**DOI:** 10.1371/journal.pone.0356357

**Published:** 2026-08-20

**Authors:** Vincent M. P. Bouchet, Fabio Francescangeli, Silvia H. M. Sousa, Márcio S. dos S. de Jesus, Jan Pawlowski, Fabrizio Frontalini

**Affiliations:** 1 Univ. Lille, CNRS, Univ. Littoral Côte d’Opale, IRD, UMR 8187, LOG, Laboratoire d’Océanologie et de Géosciences, Station Marine de Wimereux, Lille, France; 2 Department of Geosciences, University of Fribourg, Fribourg, Switzerland; 3 Instituto Oceanográfico, Universidade de São Paulo, Praça do Oceanográfico, São Paulo, São Paulo, Brazil; 4 Institute of Oceanology, Polish Academy of Sciences, Sopot, Poland; 5 ID-Gene ecodiagnostics, Confignon, Switzerland; 6 Dipartimento di Scienze Pure e Applicate, Università degli Studi di Urbino “Carlo Bo”, Urbino, Italy; University of Messina, ITALY

## Abstract

Environmental DNA (eDNA) metabarcoding has emerged as a cost- and time-efficient alternative to traditional morpho-taxonomic methods for benthic foraminiferal inventories. However, the prevalence of soft-walled monothalamous taxa of unknown ecology, which are largely underrepresented in the barcode reference database, limits the accuracy of eDNA studies. Taxonomy-free approaches, which bypass species-level assignment, may overcome these constraints. Here, we use the case study of the Armida gas platform to confirm the potential of the taxonomy-free framework for ecological quality assessment. As highlighted in a PCA, stations located near the platform (i.e., from 0 to 125 m) exhibited significantly higher concentrations of Zn and Ba in the sediment, while stations farther away appeared unpolluted. Molecular indices based on diversity, e.g., ecological quality ratio (EQR) calculated from exp(H’_bc_), and a taxonomy-free adaptation of Foram-AMBI (Foram-gAMBI) were applied, with barium serving as an independent indicator for ecological group assignment. The indices EQR (morphology), gEQR and Foram-gAMBI (eDNA) were significantly correlated with Zn and/or Ba, while Foram-AMBI (morphology) did not show any significant meaningful correlation. These results are confirmed by the inferred ecological quality statuses which revealed a clear improvement in benthic habitat quality with increasing distance from the platform when assessed with molecular indices (poor to good for gEQR and moderate to good for Foram-gAMBI), while morphological indices, particularly Foram-AMBI, failed to capture this trend. The weak agreement between morphological and molecular indices is likely due to the greater number of Molecular Operational Taxonomic Units (275) than morphospecies (31), including abundant soft-walled monothalamous foraminifera detected only by eDNA. By adopting a taxonomy-free approach to compute Foram-gAMBI, we can fully exploit the metabarcoding dataset without relying on prior taxonomic or ecological knowledge. Our findings demonstrate the robustness and accuracy of taxonomy-free metabarcoding approaches for monitoring benthic ecosystem health using benthic foraminifera in impacted marine environments.

## Introduction

Benthic foraminifera are unicellular eukaryotes largely distributed in transitional, coastal, and marine environments. As one of the most abundant meiobenthic groups, they play a key role in carbon, nitrogen, and phosphorus cycles [1–3], carbonate production [4], and bioturbation processes [5,6]. Over the last decades, they have been increasingly used as indicators of environmental changes related to human activities [e.g., 7,8–10], such as oil spills [e.g., 11], drill cutting [e.g., 12], heavy metals pollution [e.g., 13], urban sewage [e.g., 14], aquaculture [e.g., 15] and urbanization [e.g., 16].

Some biotic indices have been developed using the sensitivity of benthic foraminifera to total organic carbon [see review in 17], providing further opportunities for the application of this meiobenthic group as a biological quality element for the implementation of marine legislations [see review in 18]. Specifically, diversity [19,20], sensitivity-based [21–25] and multi-metric [26] indices have been designed for benthic foraminifera and, successfully, applied to assess the ecological quality status (EcoQS) of benthic habitats [27–32].

The foraminifera-based indices rely mostly on morphological identification of hard-shelled multi-chambered species and commonly overlook a large diversity of soft-walled single-chambered (monothalamous) taxa. Moreover, the morpho-taxonomy of some foraminiferal species is often inconsistent, hampering comparisons across studies. This issue has been raised in a meta-analysis of foraminiferal datasets from European marine and transitional waters, in which benthic foraminifera are assigned to ecological groups based on sensitivity to total organic carbon [22,24,25]. Furthermore, the widespread occurrence of cryptic species (i.e., genetically distinct yet morphologically identical) highlighted by the increasing number of molecular studies poses a major challenge for morpho-taxonomic-based biomonitoring [33]. For instance, in benthic foraminifera, morphological convergence (i.e., unrelated species showing the same morphological characteristics) or ecophenotypic variations (i.e., same species looking different under different ecological conditions) are common, making difficult to determine species by using morphology alone [34–36]. Recent studies have further suggested that different cryptic foraminiferal lineages may exhibit different ecological requirements, based on variations in spatial distribution patterns [37,38] or on meta-analyses of large datasets [25]. *In fine*, these morpho-taxonomic issues may likely hamper the implementation of biotic indices based on benthic foraminifera.

Environmental DNA (eDNA) metabarcoding is a relevant, cost- and time-saving alternative to the traditional morpho-taxonomic benthic foraminiferal inventories [see review in 39]. Studies assessing molecular alpha and beta diversity changes have shown the potential of the eDNA metabarcoding approach in monitoring the health of benthic ecosystems and its consistency with morphology‐based macrofaunal indices [40–42]. Notably, the diversity and species composition of foraminiferal assemblages inferred from eDNA metabarcoding data respond well to the impacts of aquaculture [43–45] and offshore gas and oil platform activities [46,47]. Furthermore, foraminiferal metabarcoding and morphological data are highly congruent in their diversity patterns and faunal turnover [43,47,48], confirming the accuracy of benthic foraminifera-based bioassessment. Yet, only a handful of studies have tested the use of eDNA-metabarcoding data to monitor EcoQS using benthic foraminiferal biotic indices [48–50]. For some indices (e.g., Foram-AMBI, TSI), the calculation requires a list of species assigned to ecological groups based on their responses along a pollution gradient, commonly attributed to enrichment in total organic carbon (TOC). Currently, existing lists of species only account for morphospecies [22,24,25,51]. This considerably limits the use of sensitivity-based foraminiferal indices with eDNA metabarcoding data, which are mainly composed of morphologically unassigned taxa, for ecological quality status (EcoQS) assessment.

Most metabarcoding studies of benthic foraminifera rely on taxonomic assignment to link sequences with morphospecies ecology. Yet, this strategy is severely limited by incomplete reference databases and the prevalence of soft-walled monothalamous taxa of unknown ecology, which together leave much of the metabarcoding signal unexploited. For instance, in a study on the impact of fish farming in New Zealand, only 23% of the molecular operational taxonomy units (MOTUs) could be assigned to a morphospecies and used to calculate Foram-AMBI (called metabarcoding biotic index in the study) to monitor the EcoQS of soft-sediments [52]. It means that about 77% of the biological signal in the foraminiferal eDNA metabarcoding data was not considered, resulting in EcoQS characterizations that may not give an accurate picture of the health of the studied benthic habitats. Unless these constraints are overcome, eDNA-based biomonitoring will fall short of its potential to provide robust evaluations of marine ecosystem health.

Taxonomy-free metabarcoding approaches, where eDNA data are used but not assigned to specific taxonomic classes, may overcome this limitation. Specifically, the morphologically unidentified OTUs are assigned to particular ecological conditions to monitor the EcoQS. The usefulness of this approach was first demonstrated in the context of benthic diatoms used to monitor the ecological quality of rivers and streams [53,54], nematodes [55] and with benthic foraminifera [49,56]. In general, these works either use a leave-one-out cross-validation procedure or split their dataset in two, i.e., a calibration dataset used to assign MOTUs or ASVs to ecological group and a test dataset to evaluate the performance of the calibration. Further advances in taxonomy-free metabarcoding combined with supervised machine learning have led to very promising results for monitoring changes in benthic communities in marine aquaculture [57] and, more generally, for routine ecosystem monitoring using environmental genomics [58,59].

In this context, we investigated the possibility of using a taxonomy-free approach for computing of Foram-AMBI directly from eDNA data without any reference to morphospecies. The present study focuses on the impact of the offshore gas platform “Armida” located in the Northern Adriatic Sea (Italy). This study is part of a comprehensive research project aiming at assessing the impact of this platform on the benthic communities, i.e., macro-invertebrates and foraminifera by mean of morpho-taxonomy and eDNA metabarcoding [41,47]. It was reported that barium (Ba) concentration is a good indicator of the impact of such anthropogenic structures on benthic habitats and macrofauna [60,61]. Therefore, we used Ba concentrations as the environmental driver to assign MOTUs to ecological groups. The aims of this study are (i) to apply a taxonomy-free approach on eDNA metabarcoding data using a leave-one-out cross validation procedure to compute the sensitivity index Foram-gAMBI [53], (ii) to consider potential correlations with environmental parameters of the diversity index Exp(H’_bc_) and Foram-AMBI index calculated with traditional morpho-taxonomy and eDNA metabarcoding data, and (iii) to compare morpho-taxonomy and eDNA-based EcoQS. We hypothesize that the taxonomy-free approach would perform better at monitoring the EcoQS as it is not biased by difficulties with morphological identification or limited to taxa present in genomic reference databases.

## Materials and methods

### Sampling design

**Fig 1:**
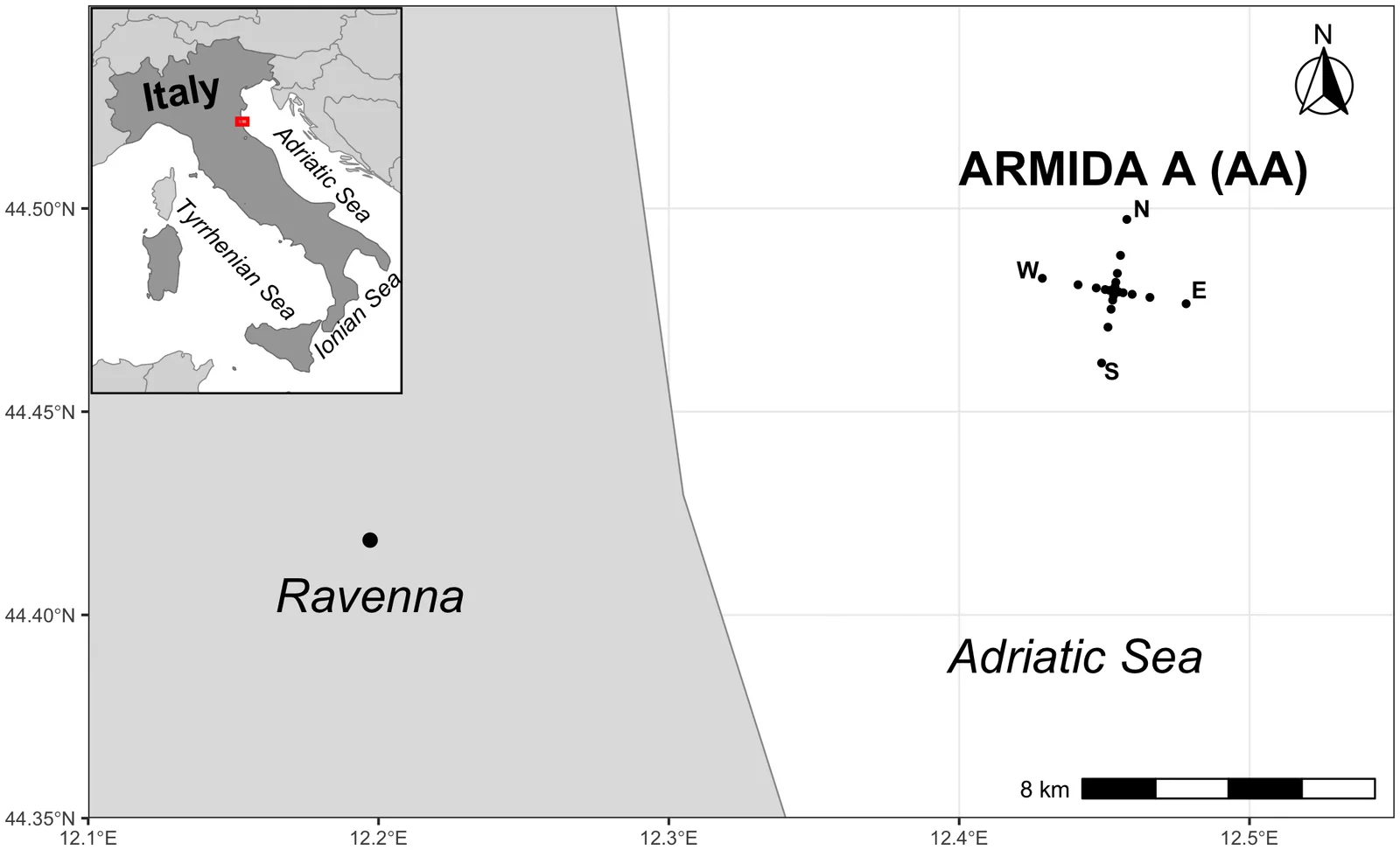
Map of the sampling sites in Italy in front of the city of Ravenna in the northern part of the Adriatic Sea. W: West, N: North, E: East, and S: South.

Ninety-six sediment samples were collected with a box-corer around the Armida gas platforms in 2017 [for more details, see 47]. Samples were collected along four transects (North, West, South, and East) and at increasing distances (0 m, 25 m, 50 m, 125 m, 250 m, 500 m, 1000 m, and 2000 m) from the platform (Fig 1). Three replicates (A, B and C) from independent box-corer deployments were collected at each station, and only the uppermost part of the sediment (0–1 cm) was retained for analyses. For the foraminiferal morphological analyses, immediately after sampling, sediments were placed in Falcon tubes and treated with a rose Bengal solution (2 g of rose Bengal in 1000 ml of ethanol) for at least 14 days to distinguish living from dead foraminifera, then gently mixed. Approximately 10 g of surface sediment (0–1 cm) was collected using a sterile spoon for eDNA metabarcoding. The sediment samples were preserved in LifeGuard^TM^ Soil Preservation Solution (Qiagen) and immediately frozen at −20°C. Additional aliquots of sediment were collected and immediately frozen for subsequent grain-size, organic matter, and geochemical analyses.

#### Sediment analyses.

Detailed descriptions of grain-size and geochemical analyses, including total organic carbon (TOC) and organic and inorganic contaminants are reported in Cordier et al. [41] and Frontalini et al. [47]. Synthetically, sediments for grain-size analysis were treated with an H_2_O_2_ solution, sieved, and dried at 40°C, then analyzed using a series of ASTM micro-sieves (>63 µm) and a Sedigraph (< 63 μm).

The TOC, trace elements, mercury, polycyclic aromatic hydrocarbons (PAHs), aliphatic hydrocarbons, and other hydrocarbons were analyzed following the U.S. EPA 6020B (2014), U.S. EPA 7474 (2007), U.S. EPA 8270D (2014), U.S. EPA 8260C (2006), and ISO 16703 (2004) methods, respectively. Only Ba and Zn were considered in the analyses, as these elements reached high levels with potential harmful effects.

### Foraminiferal analyses

#### Morphological identification.

Sediment samples were dried at 50 °C and weighed, then gently washed through a 63 μm sieve with tap water to remove clay, silt, and any excess dye. Quantitative analyses were performed on the fractions >63 μm from all replicates by hand-picking living (i.e., rose-Bengal stained) benthic foraminiferal specimens. Specimens were taxonomically identified essentially following Frontalini and Coccioni [62 and reference therein].

#### eDNA extraction, PCR amplification and high-throughput sequencing.

The full method is detailed in Frontalini et al. [47]. In short, sediment samples were extracted using the DNeasy Power Soil Kit (Qiagen). For each sediment sample, three extractions were performed. For each extraction, 0.5 ml of sediment were treated according to the manufacturer’s instructions with bead beating at 6.5 m/s for 45 s in a high-speed homogenizer (MP biomedicals). All extractions were performed in a PCR-free environment, with blank extractions (i.e., extraction assay without sediment) included to control for (cross-) contamination events. A DNA fragment encompassing the foraminiferal-specific 37f and 41f hypervariable regions of the 18S rRNA gene was PCR-amplified from each eDNA extract, using foraminiferal-specific primers s14F1 (5′AAGGGCACCACAAGAACGC) and s17 (5′CGGTCACGTTCGTTGC) [63]. The amplified fragment was about 350 base pairs long. Tagged primers bearing eight nucleotides attached at each primer’s 5’-extremity were used to enable multiplexing of all PCR products in a unique sequencing library [64]. The pooled replicate PCR products for each sample were quantified by high-resolution capillary electrophoresis using QIAxcel System (Qiagen). Equimolar concentrations of PCR products were pooled for each library. Each library was purified using High Pure PCR Product Purification kit (Roche Applied Science) and the libraries preparation was performed using Illumina TruSeq® DNA PCR-Free Library Preparation Kit. The libraries were then quantified with qPCR using KAPA Library Quantification Kit and sequenced on a MiSeq instrument using paired-end sequencing for 600 cycles with kit v3 (Illumina). Molecular analyses, including sequencing, were conducted in ID-Gene ecodiagnostics (Switzerland). The raw data are available from the Sequence Read Archive public database under the accession PRJEB29469.

#### Bioinformatics.

As described in Frontalini et al. [47], raw sequencing data were quality-filtered by removing any sequence with a mean quality score of 30, and also removing all sequences with ambiguous bases or any mismatch in the tagged primer. These extremely stringent parameters ensure that we keep only high-quality data. Then, paired-end reads were assembled by aligning them into a contiguous long-length sequence. Chimera removal, strict dereplication, and the Molecular Operational Taxonomic Units (MOTUs) clustering were performed using the SWARM v2.1.8 algorithm with the default d parameter (i.e., 1) [65]. The total number of raw sequences is 17,177,281, of which 5,067,597 are retained for downstream analysis after stringent quality filtering. (Frontalini et al. 2020). Overall, 22,886 MOTUs are produced by the SWARM clustering algorithm. Then, MOTUs’ representatives were compared against a curated reference sequence database for taxonomic assignments [66] using the assign_taxonomy.py script from the QIIME v1.9.1 toolkit [67] with the default option [uclust method, 68]. Annotations were performed using the Last Common Ancestor approach from up to three candidate reference sequences with above 95% similarity with the queries. To remove the noise in our data due to the presence of rare genetic variants or eDNA molecules transported from other areas, which could influence the alpha diversity analysis, we filtered out the MOTUs represented by less than 1000 reads. This reduced the number of MOTUs to 343 [data available in 47]. Among them, 84 and 27 MOTUs are assigned to single-chambered Monothalamea and multichambered calcareous or agglutinated Globothalamea, respectively, whereas 232 MOTUs remain unassigned (Frontalini et al. 2020). To facilitate the comparison of molecular and morphological data, the MOTUs assigned to the same morphospecies have been combined, reducing the total number of assigned MOTUs to 43.

### Ecological quality status assessment

#### Diversity indices.

Diversity indices were calculated at the replicate level for two datasets, i.e., (i) morphological, and (ii) the complete eDNA. Species richness was computed from presence–absence matrices, while Exp(H’_bc_) was calculated from raw data. Station-level values represent means ± standard deviation across three replicates.

The diversity index Exp(H’_bc_) was also used to assess the EcoQS [20]. Note that a “g” prefix (for genetic) was added for the molecular version used in the present study. The EcoQS class boundaries have not been defined yet for the gExp(H’_bc_). Because molecular diversity is much higher than morphological diversity, class boundaries developed for morphological datasets [20] cannot be applied. Therefore, we used the ecological quality ratio (EQR), as suggested in the European Water Framework Directive (WFD, 2000/60/EC). The EQR is the ratio between the value of a biological metric, such as Exp(H’_bc_) or gExp(H’_bc_), and the expected value under reference conditions [69]. The EQR or gEQR varies, therefore, between 0 (i.e., bad EcoQS) and 1 (i.e., high EcoQS). Local-specific reference conditions are the anchor points for calculating the EQR and gEQR; here, we used the stations’ 95^th^ percentile of Exp(H’_bc_) and gExp(H’_bc_), respectively, as reference conditions and followed the WFD recommendations for the EcoQS boundaries [69, Table 1]. This conservative approach appears robust to determine true reference conditions as stations farther away from the platform exhibits low levels of Ba and Zn, indicating that they are not polluted (S1 Table).

**Table 1:** Criteria for determining EcoQS according to Ba (see above in the section describing the leave-one-replicate-out procedure), the EQR and gEQR [69], and Foram-AMBI and Foram-gAMBI [70].

EcoQS and associated colour code	Bad	Poor	Moderate	Good	High
Barium (in sediment, mg.kg^-1^)	>1000	540-1000	210-540	133-210	<133
EQR and gEQR	<0.25	0.25-0.5	0.5-0.7	0.7-0.9	>0.9
Foram-AMBI, ass-Foram-gAMBI and Foram-gAMBI	>4.4p	3.4-4.4	2.4-3.4	1.4-2.4	<1.4

#### Sensitivity index.

The sensitivity index Foram-AMBI was used in this study (Alve et al. 2016). The calculation of Foram-AMBI requires assigning species to ecological groups (EGs) based on the responses of foraminiferal species along a pollution gradient, typically TOC [22]. For the morphological dataset, the species’ assignments for foraminiferal species from open waters in the Mediterranean Sea were used [24] and are presented in S2 Table. The assignments to EG for eDNA data are presented in detail in the section describing the leave-one-replicate-out procedure. For Foram-AMBI and Foram-gAMBI, we used EcoQS boundaries developed in open waters in the Mediterranean Sea [70, Table 1].

#### MOTUs assignment to ecological groups.

**MOTUs with corresponding morphospecies:** Some of the MOTUs could be assigned to a morphospecies. Therefore, existing classifications in ecological groups from morphological [24,71] and molecular [43,56] were used to find the ecological groups of MOTUs assigned to a morphospecies, hereafter called the assigned MOTUs eDNA dataset (S3 Table). This was used to calculate ass-Foram-gAMBI (S3 Table).

**Leave-one-replicate-out taxonomy free procedure for MOTUs without a correspondence to a morphospecies:** Most MOTUs recorded in the present study did not correspond to a morphospecies due to incomplete reference databases; hence, there was no corresponding ecological group for Foram-gAMBI calculation. We therefore developed a tailored list for MOTUs recorded in the present study using a taxonomy-free approach. To avoid bias and ensure consistency, MOTUs corresponding to morphospecies were also included in the new tailored list (S4 Table).

To limit self-calibration bias and evaluate predictive robustness, a leave-one-replicate-out cross-validation procedure was implemented across all stations. Replicates (A, B, and C) were used in this framework, where replicates sharing the same identity (A, B, or C) were successively held out across all stations, while the others were used for training; ecological optima were estimated from the training replicates and applied to the held-out group for Foram-gAMBI calculation. The three calibration–application cycles were as follows:

- Training datasets for Replicate A MOTUs assignment to EGs: Replicates B + C (all stations) → MOTUs optima estimation → Assignment to EGs → Foram-gAMBI predicted for replicate A at each station
- Training datasets for Replicate B MOTUs assignment to EGs: Replicates A + C (all stations) → MOTUs optima estimation → Assignment to EGs → Foram-gAMBI predicted for replicate B at each station
- Training datasets for Replicate C MOTUs assignment to EGs: Replicates A + B (all stations) → MOTUs optima estimation → Assignment to EGs → Foram-gAMBI predicted for replicate C at each station

In each cycle, samples used for index calculation were not included in optimum estimation (S4 Table). This procedure generates out-of-fold replicate-level predictions while preserving the full station set in each iteration. Because sediment replicates originate from the same station and share identical environmental values, the procedure controls replicate-level circularity but does not constitute full ecological independence at the station level.

Three replicate-level predicted Foram-gAMBI values obtained from the leave-one-replicate-out procedure were averaged to compute a single station-level index. Performance of the leave-one-replicate-out procedure was assessed by quantifying the association between station-level predicted Foram-gAMBI values and measured barium concentrations using Kendall’s rank correlation coefficient.

In the case of an offshore gas platform, Ba concentrations have been reported as a good indicator of the impact of such anthropogenic structures on benthic habitats and fauna [60,61]. Unfortunately, the criteria for evaluating EcoQS with Ba were unavailable in the literature [72]. We therefore decided to develop a set of criteria for this purpose. According to the literature, the safety limit in sediment is a Ba concentration of 133 mg.kg^-1^ [73], and the mean natural abundance was reported as 207.4 mg.kg^-1^ [74]. The threshold to detect harmful effects is at 400–550 mg.kg^-1^ [73,75], and the EC50 value was observed at about 1000 mg.kg^-1^ [75]. Based on these findings, we therefore established the following criteria for evaluation of EcoQS with Ba in sediment (in mg.kg^-1^): < 133: high EcoQS, 133–210: good EcoQS, 210–540: moderate EcoQS, 540–1000: poor EcoQS and >1000: bad EcoQS (Table 1).

Therefore, we used Ba concentrations to assign MOTUs to the five EGs of Foram-gAMBI. The weighted-averaging (WA) optimum [76,77] was computed for MOTUs to determine their response to Ba, following the method of Bouchet et al. [25], and was successfully tested on MOTUs in Cavaliere et al. [49]. A simple and ecologically reasonable estimate of a benthic foraminiferal species’ optimum is the average of all Ba values for the present study sampling stations in which the MOTUs occur, weighted by the MOTUs’ relative abundance (WA regression).

*In fine*, the next two steps were applied to assign foraminiferal MOTUs to one of the five EGs. To do so, as described in the methods [25,51], the criteria used to evaluate EcoQS for Ba in sediments developed in the present study (see above) were applied to assign the MOTUs in the EGs. First, the WA optimum was computed for each MOTU. Secondly, MOTUs’ assignment to EGs was done as follows: if a MOTU had an optimum in the Ba range 0−133 mg.kg^-1^, the MOTU was assigned to EGI; in the range 133−210 mg.kg^-1^, the MOTU was assigned to EGII; in the range 210−540 mg.kg^-1^, 540−1000 mg.kg^-1^, and above 1000 mg.kg^-1^to EGIII, EGIV, and EGV, respectively, based on Ba-derived EcoQS (Table 1).

### Statistical analysis

A Principal Component Analysis (PCA) was performed on environmental variables. Prior to the PCA, we conducted a covariance analysis to identify and exclude highly collinear variables. Specifically, pairwise Pearson correlation coefficients were computed, and variables with absolute correlation values exceeding 0.9 were removed to avoid redundancy. Remaining variables exhibiting strong right-skewness (Ba, Zn, silt, sand and TOC, S1 Table) were log-transformed using log(x + 1), then centered and scaled to unit variance. A principal component analysis (PCA) was performed on the standardized variables.

To assess directional trends in diversity, analyses were conducted separately for each transect (North, South, East, West). For each transect, the relationship between diversity metrics and distance from the origin was evaluated using regression models with log-transformed distance (log(distance + 1)) as a continuous predictor. Species richness (count data) was analysed using generalized linear models (GLMs) with a log link function and either Poisson or negative binomial error distributions, depending on the presence of overdispersion. The exp(H’_bc_), treated as a continuous variable, was analyzed using linear models (LMs) with Gaussian error distribution. For each transect and response variable, the slope of the relationship with log-transformed distance was estimated, and its significance was assessed using Wald tests (z-tests for GLMs and t-tests for linear models).

Correlation between indices and environmental parameters was investigated using Kendall’s coefficient of rank correlation (*τ*). Kendall’s coefficient of correlation was used in preference to Spearman’s coefficient of correlation (*ρ*), because Spearman’s *ρ* gives greater weight to pairs of ranks that are further apart, while Kendall’s *τ* weights each disagreement in rank equally [78].

To analyze the agreement between biotic indices used to assess EcoQS, a Kappa analysis was conducted [79,80] following Borja et al. [81]. The level of agreement between indices was established based on the equivalence table from Monserud and Leemans [82]. As the probability of misclassification is not the same between close categories (e.g., high and good, or poor and bad) as between distant categories (e.g., high and moderate, or high and bad), Fleiss–Cohen weights were applied to the analysis [83].

All statistical analyses were done with the R software v4.5.2 [84].

## Results

### Environmental characterization

**Fig 2:**
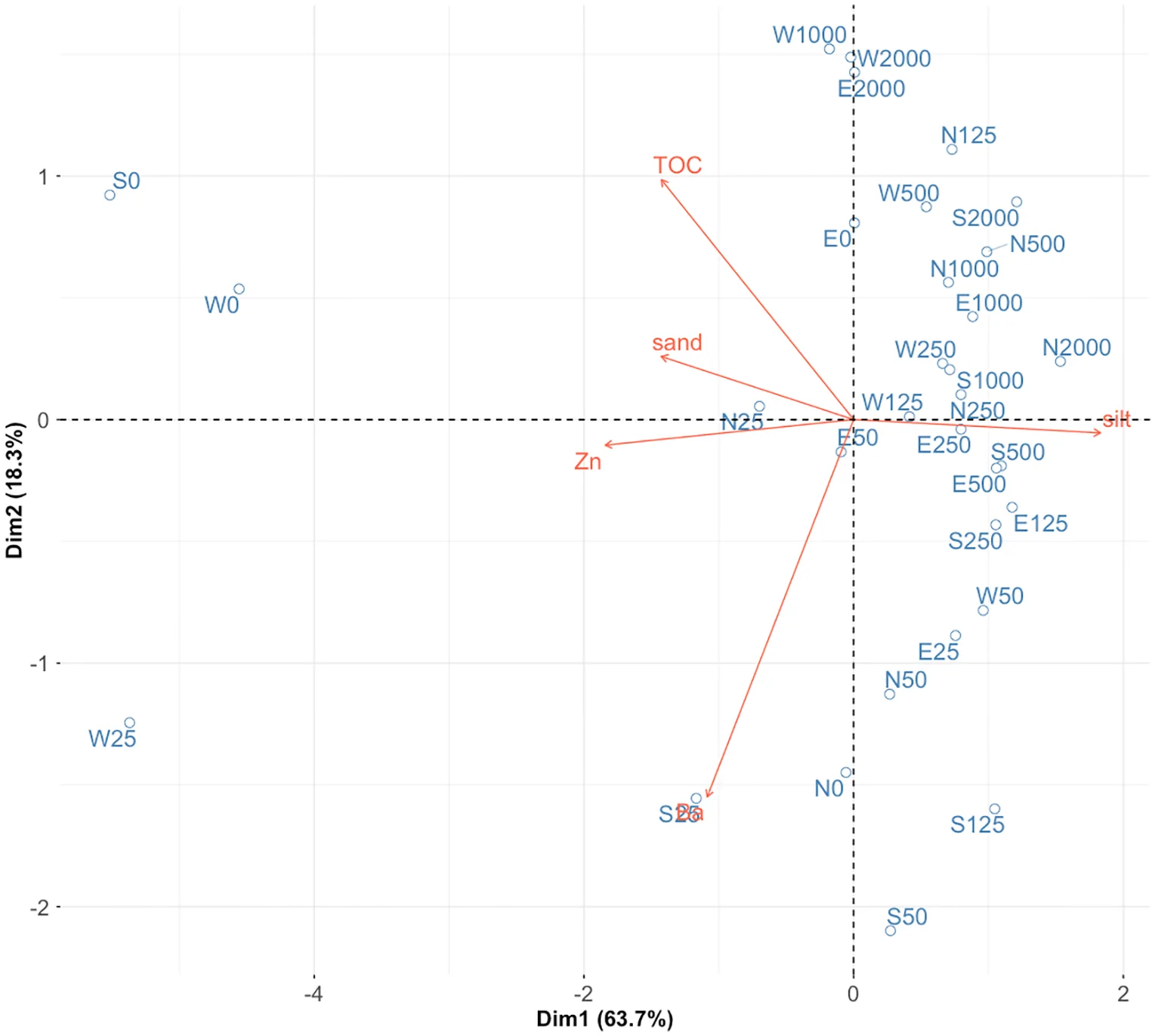
Principal component analysis of selected environmental parameters. (W: West, N: North, East: East and S: South with 0, 25, 50, 125, 250, 500, 1000 and 2000 being distance in m from the platform).

The PCA analysis of selected environmental parameters from the Armida platform showed that two principal axes accounted for 82% of the total variance (Fig 2). The top contributors to the first axis included variables such as Zn, sand and silt that together explained 63.7% of the variability observed across sampling stations (Fig 2). In turn, the second axis explained about 18.3% of the variability, with top contributors being TOC and Ba. These results indicated that the first axis is mostly driven by the sediment physical features, i.e., grain size and to a certain extent by Zn; and the second axis primarily reflects a “pollution gradient” driven by Ba contamination in the sediment. Most of the stations located near the platform (i.e., from 0 to 125 m) showed a positive correlation with Ba and Zn, while stations farther away were negatively correlated.

### Diversity assessment

**Fig 3:**
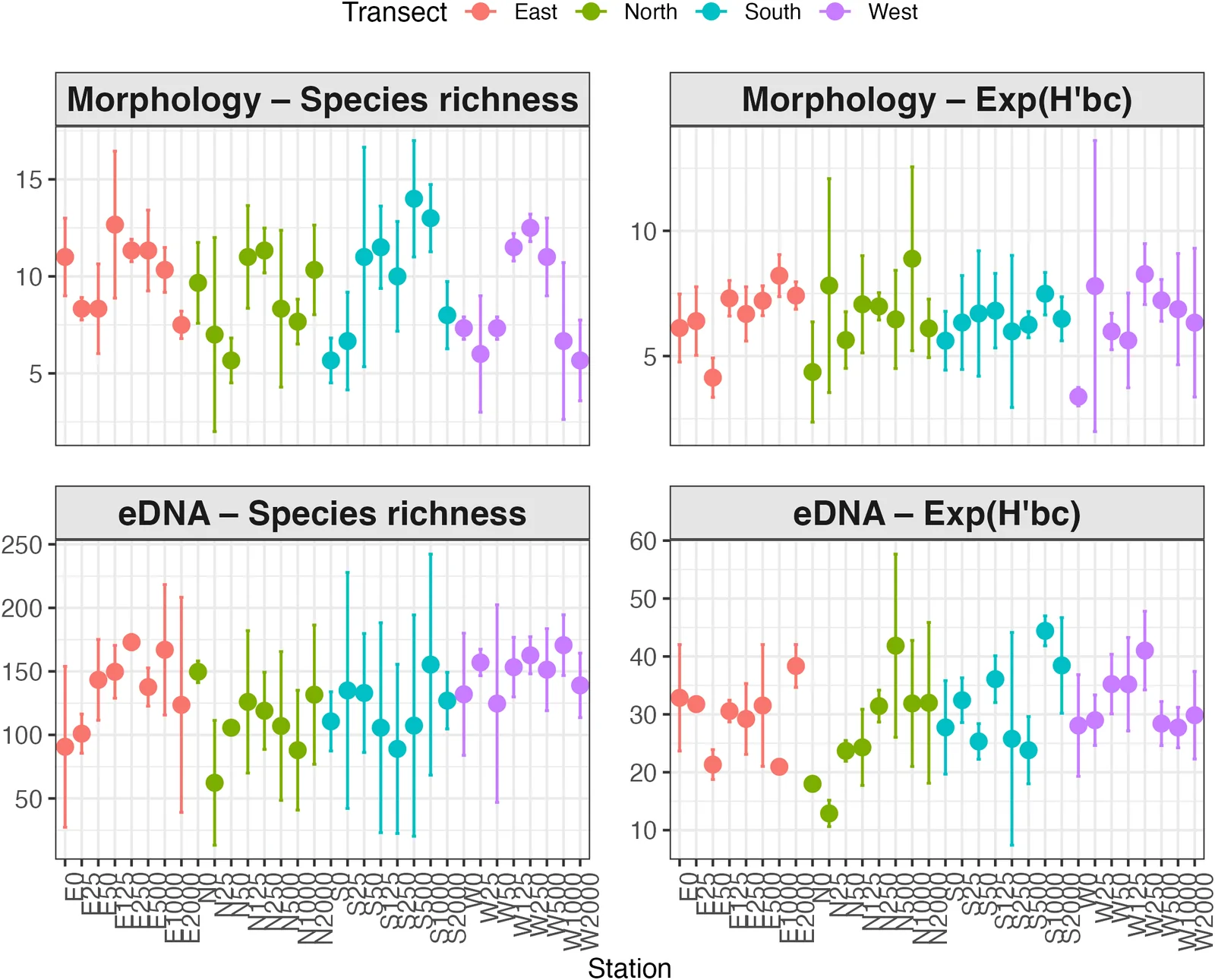
Species richness and Exp(H’_bc_) at each station for the morphology and eDNA datasets (mean±sd). (W: West, N: North, East: East, and S: South with 0, 25, 50, 125, 250, 500, 1000, and 2000 being distances in m from the platform).

A total of 31 species were identified in the morphological samples (S2 Table). Species richness values varied between 5.6 ± 2 and 14 ± 3 (Fig 3) and Exp(H’_bc_) between 3.3 ± 0.3 and 8.2 ± 1.2 (Fig 3). There is no trend along the distance gradient from the platform in terms of species richness (GLM with Poisson errors, *p* > 0.05), apart along the South transect which exhibit a significant positive relationship with log-transformed distance (GLM with Poisson errors, estimate = 0.087, *p* < 0.01), indicating an increase in species richness with distance. Regarding Exp(H’_bc_), there was no trend along the South, West and North transect (LM, *p* > 0.05), while it significantly increased with distance along the East transect (LM: slope = 0.279, *p* < 0.05).

Diversity was higher in the eDNA dataset. In detail, 275 MOTUs were retrieved (S4 Table), species richness ranged between 62.3 ± 49.1, and 173 ± 6 (Fig 3), and gExp(H’_bc_) between 12.8 ± 2.2 and 44.4 ± 2.5 (Fig 3). No trend was observed along the distance gradient from the platform in terms of species richness (GLM with negative binomial errors, *p* > 0.05). Diversity estimated with gExp(H’_bc_) tended to significantly increase along the North transect (LM: slope = 2.84, *p* < 0.01), while no significant trends were observed in the other directions (LM, *p* > 0.05).

### Biotic indices and environmental parameters

Only 43 out of the 275 MOTUs could be used to compute ass-Foram-gAMBI on MOTUs assigned to a morphospecies (S3 and S4 Tables), which involved considering, on average, 44.7 ± 11.2% of reads per replicate (min = 16%, max = 78%, S3 Table). This value dropped to 38.5 ± 13.3% if we considered only MOTUs assigned to a morphospecies and also to an ecological group for ass-Foram-gAMBI calculation. Furthermore, a total of 23 replicates had values of not assigned (NA) species to an EG between 21 and 86%; ass-Foram-gAMBI was therefore not calculated for these replicates, as these values are above the 20% threshold [85]. No value of the index was recovered for the following stations: West 0, 25, and 2000m, East 50m (S1 Fig). The index ass-Foram-gAMBI was not correlated to any environmental parameters (S2 Fig).

In the calculation of Foram-AMBI and Foram-gAMBI, the number of unassigned species or MOTUs to EGs was between 0–14% (S2 Table) and 0–1% (S4 Table), respectively. The reader may note that, for the morphological data, replicate North 1 at 25 m away from the platform and replicate North 3 at 500 m away from the platform had, respectively, 33% and 44% of unassigned species and were, therefore, not considered for the calculation of Foram-AMBI as these values are above the 20% threshold [85].

**Fig 4:**
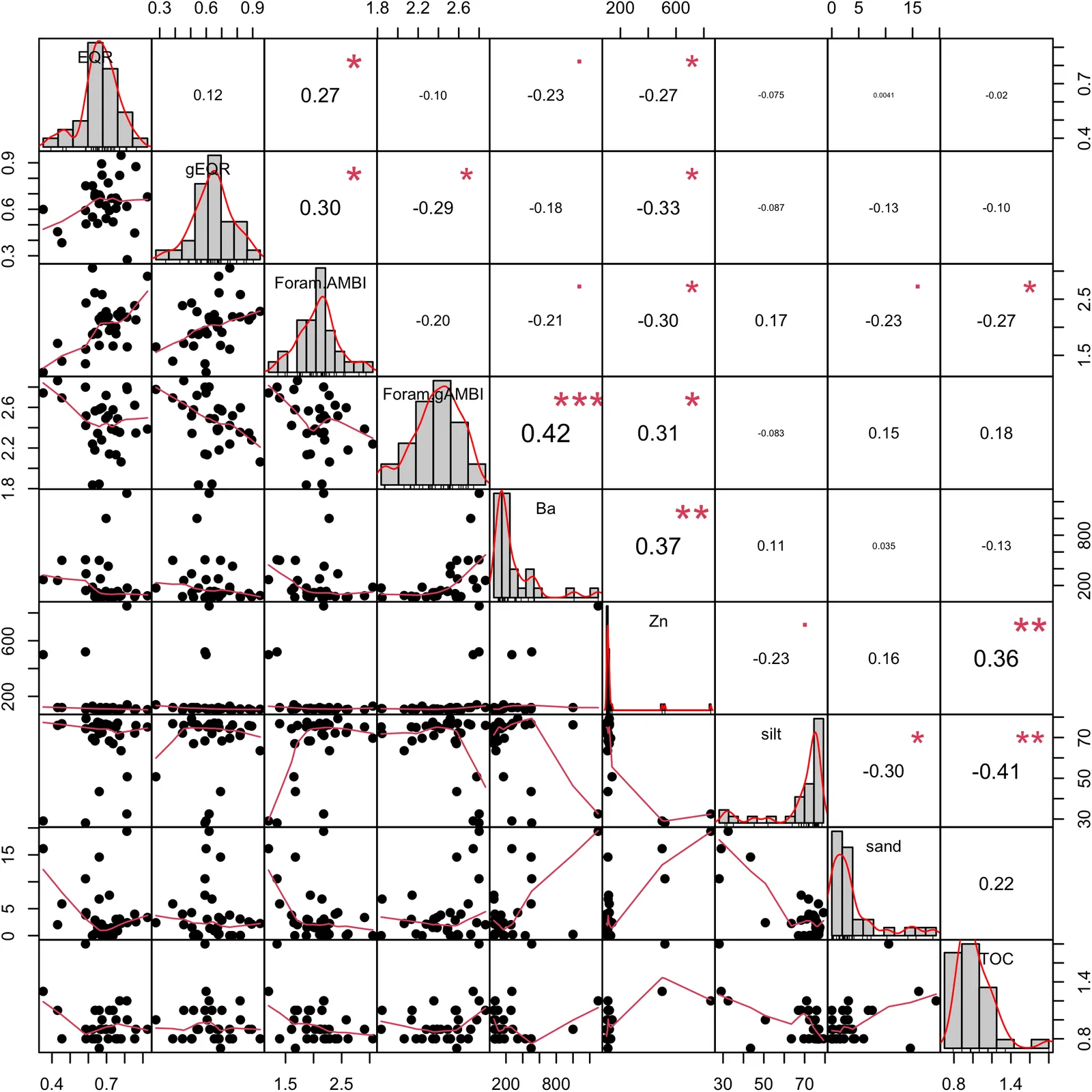
Correlation between biotic indices and environmental parameters. Kendall’s coefficient of rank correlation (*τ*) is reported. Significant correlations are indicated: *: *p* < 0.05, **: *p* < 0.01, ***: *p* < 0.001.

Regarding indices based on morphological data, EQR was significantly correlated negatively with Zn (Kendall, *τ* = −0.27, *p* < 0.05, Fig 4), while Foram-AMBI was significantly correlated negatively with Zn and TOC (Kendall, *τ* = −0.30 and *τ* = −0.27, *p* < 0.05, Fig 4). For the molecular-based indices, correlation between gEQR and Zn was negatively significant (Kendall, *τ* = −0.33, *p* < 0.05, Fig 4). The index Foram-gAMBI was significantly positively correlated with Ba (Kendall, *τ* = 0.42, *p* < 0.001, Fig 4) and Zn (Kendall, *τ* = 0.31, *p* < 0.05, Fig 4).

### Ecological quality status around the gas platform based on benthic foraminifera

**Fig 5:**
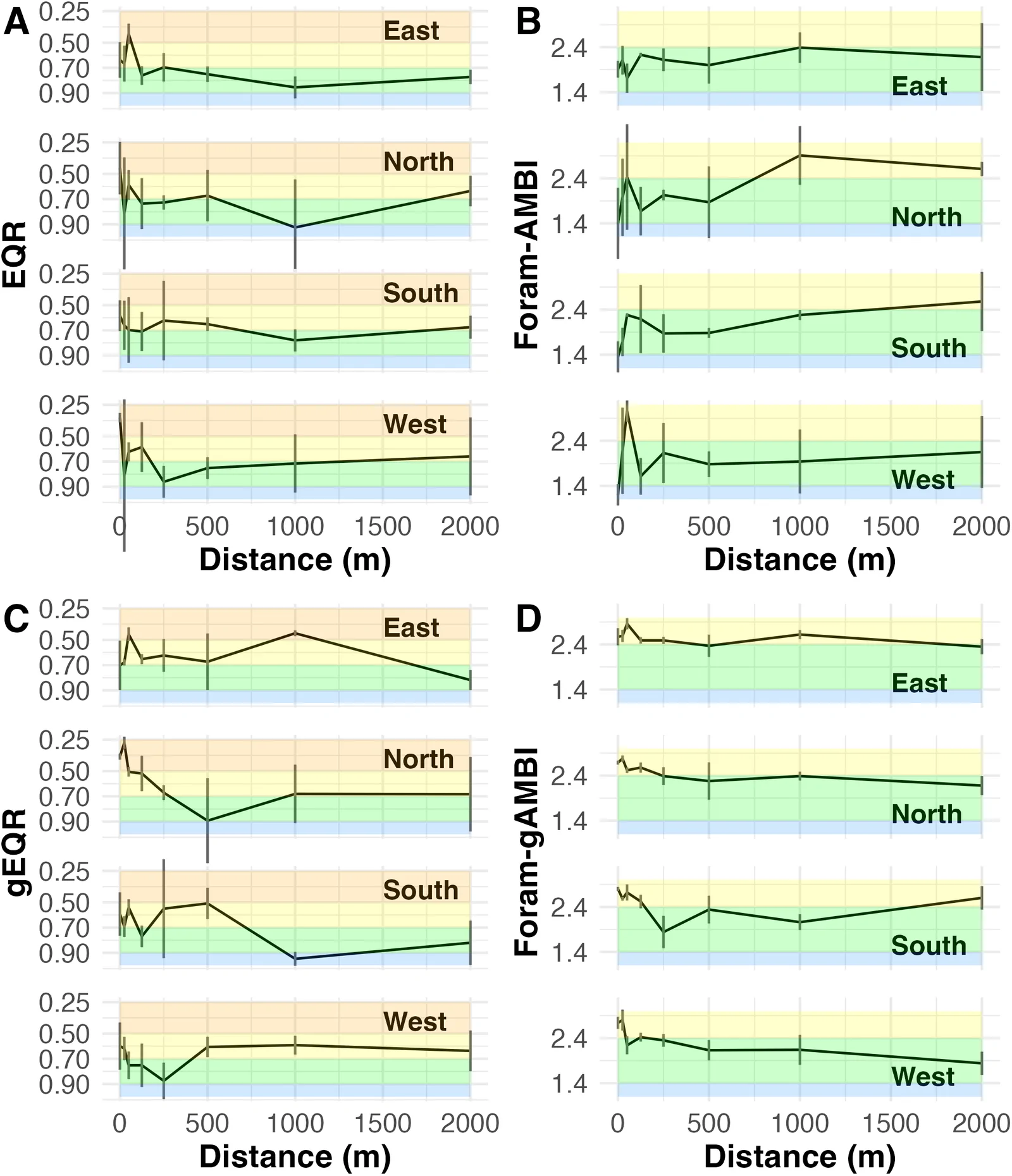
Values (mean ± s.d.) of indices based on morphological foraminiferal identification (A: EQR and B: Foram-AMBI) and on eDNA data (C: gEQR and D: Foram-gAMBI) along the four transects (East, North, South, and West) of the Armida gas platform. Corresponding EcoQS are reported (High: blue, Good: green, Moderate: yellow, and Poor: orange).

Based on the morphological diversity index, the EcoQS tended to improve from moderate to good with EQR until 1000 m, and then it degraded towards moderate at 2000 m except for the East transect (Fig 5A). Conversely, EcoQS worsened from good to moderate with Foram-AMBI from 0 to 2000 m along the South and North transects, while the East and West transect did not show any trend (Fig 5B).

Following gEQR, EcoQS varied between poor and moderate near the platform, and from moderate to good far from it, apart from the West transect which exhibited moderate EcoQS at 0, 50, 500, 1000 and at 2000m from the platform (Fig 5C). The Foram-gAMBI classified as moderate EcoQS stations in the vicinity of the platform (from 0 to 250 m) and as good at the most distant stations, apart at 2000m from the platform along the South transect (Fig 5D). Both molecular indices suggested an improvement in the EcoQS from 0 to 2000 m away from the platform along most of the transects.

### Correlation between indices and level of agreement

The morphological indices EQR and Foram-AMBI were positively correlated (Kendall, *τ* = 0.32, *p* < 0.01, Fig 4). Conversely, the molecular indices were negatively correlated (Kendall, *τ* = −0.29, *p* < 0.05, Fig 4). There was no significant correlation between the morphological and molecular indices (Kendall, *p* > 0.05, Fig 4).

**Fig 6:**
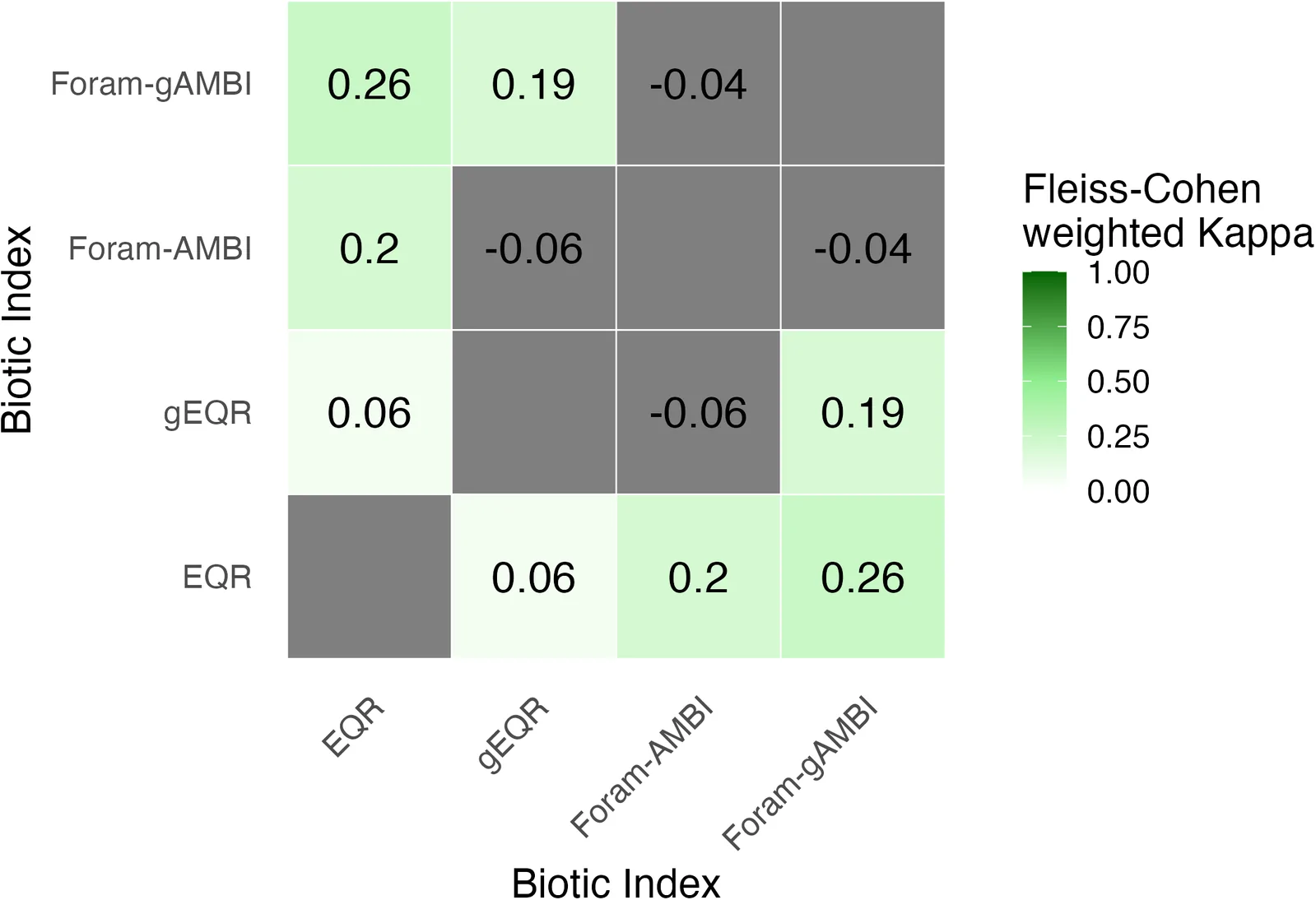
Kappa values and agreement for the EcoQS analysis (in color) between the different biotic indices used in this study.

There was a fair agreement in terms of EcoQS classification between EQR and Foram-gAMBI (Kappa, κ = 0.26, Fig 6). Kappa statistics showed a slight agreement between EQR and Foram-AMBI (Kappa, κ = 0.2, Fig 6), gEQR and Foram-gAMBI (Kappa, κ = 0.19, Fig 6) and EQR and gEQR (Kappa, κ = 0.06, Fig 6).

## Discussion

Environmental DNA-based biomonitoring with benthic foraminifera is sometimes challenged by the limitations of taxonomic reference databases and the limited ecological knowledge available for most metabarcoding data. In addition, eDNA datasets can be affected by the presence of DNA molecules of extraneous origin (e.g., transported and preserved in the sediment), which may constitute background noise. To limit this, a common approach is to filter out low-abundance MOTUs, since this exogenous DNA generally occurs at lower abundances. This filtering step also removes rare genetic variants resulting from intragenomic polymorphism, which is common in the ribosomal operons of foraminifera. Although the filtration threshold is arbitrary, we chose 1000 reads to ensure that the analysed data corresponded to the local population actually living *in situ*. We consider this step essential for the robustness of metabarcoding data in ecological studies.

Beyond this filtering step, taxonomy-free metabarcoding offers additional advantages: by not assigning eDNA reads to specific taxonomic classes, this approach is not reliant on accurate taxonomic identification and is not limited by inadequate reference database coverage. Furthermore, eDNA metabarcoding takes relatively less time than morphological foraminiferal identification and counting. Thus, applying taxonomy-free on foraminiferal data provides a rapid, standardized, and simplified method for assessing the ecological quality status of estuarine and marine ecosystems [e.g., 49,56]. In the present study, we tested three approaches: morphological, MOTUs assigned to morphospecies and taxonomy-free. We only reported a significant correlation with observed barium concentrations in sediment for the last approach. This result supports the growing evidence that taxonomy-free metabarcoding presents a robust and scalable method for environmental biomonitoring [53,55,86].

### Taxonomy-free approach overcomes the lack of ecological knowledge

This study implements a taxonomy-free approach for EcoQS assessment with eDNA foraminiferal indices in the context of gas production in the Adriatic Sea. This approach allows us to produce a list of MOTUs and their corresponding ecological group assignments tailored to the observed pollution gradient (i.e., barium enrichment) typically observed near offshore gas platforms [60,61]. The use of part of the dataset for calibrating the assignment to ecological group was mandatory to limit circular reasoning which could have severely biased our work. Alternative approaches could have been considered, such as a leave-one-station-out procedure (53) or machine-learning-based methods (57). We reasoned that working at the replicate level (i.e., leave-one-replicate-out) allowed us to capture the full Ba gradient represented in this dataset, rather than grouping replicates into discrete Ba concentration classes, which would have reduced the resolution of the gradient and introduced arbitrary class boundaries. This approach therefore provided a more robust calibration and, consequently, a more reliable assignment of MOTUs to ecological groups for Foram-gAMBI calculation.

On average, we lost about 61.5% of the biological signal in the molecular dataset when ass-Foram-gAMBI was calculated using only the MOTUs assigned to morphospecies with a known ecological group. The ass-Foram-gAMBI did not correlate with any of the environmental parameters, further exhibiting the poor accuracy of using only assigned MOTUs. Furthermore, the use of different case studies spanning from Norway to the Mediterranean Sea to find corresponding EG for each assigned MOTU does not account for possible ecological shifts between geographical areas [25,51,87] and may explain the limitations of ass-Foram-gAMBI in the present study. The lack of knowledge on monothalamous species ecological requirements also hampers the implementation of eDNA for biomonitoring [48,56]. In the assigned MOTUs eDNA dataset, 100% of the MOTUs that could not be assigned to any EG belonged to monothalamous morphospecies, accounting for 13 of the 31 monothalamous morphospecies identified in the present study, thereby excluding an important proportion of the biological signal from the EcoQS assessment. This further highlights that more studies on the environmental drivers of monothalamous species distribution patterns are required, as they represent a substantial number of species in benthic foraminiferal communities.

The taxonomy-free approach appeared as the only solution in our case, due to the dominance of monothalamous foraminifera in metabarcoding datasets. Even if some of them could be assigned to the reference database, only few of them have been assigned to ecological groups [52,71]. The poor ecological knowledge of monothalamids practically excludes them from being used as bioindicators or to include them into ecological indices such as Foram-gAMBI. As shown by our study, the taxonomy-free approach overcomes this limitation and enables us to use the whole MOTUs dataset. Indeed, the main benefit of the taxonomy-free approach lies in its much higher data coverage compared to the use of taxonomic assignment [53]. The implementation of Foram-gAMBI did not require taxonomical or ecological knowledge to assign MOTUs to EGs.

The taxonomy-free approach in biomonitoring surveys based on eDNA metabarcoding is increasingly acknowledged as a promising method for routine EcoQS assessment [see review in 88]. It is important to note that the method presented here is one possible variant of the taxonomy-free approach applied to biomonitoring. An alternative is to use Supervised Machine Learning (SML) algorithms to predict Biotic Index (BI) values (Cordier et al. 2021). These SML algorithms develop predictive models from complex datasets by considering the entire community and accounting for the co-occurrence of MOTUs. It requires training datasets with samples of known EcoQS to define BI. In the present study, the Foram-gAMBI EcoQS status was instead calibrated directly against stressor values (e.g., Ba) using a leave-one-replicate-out cross-validation procedure in line with the very few foraminiferal studies which tested the taxonomy-free approach [49,56]. This direct calibration allows us to provide a more straightforward interpretation of the ecological impact of the Armida platform, potentially offering a more precise assessment of environmental quality. However, this study is one of the few to use taxonomy-free eDNA-based foraminiferal indices, and as such, it is necessary to further validate its application. This has to be done through intercalibration exercises in different ecosystems and including different environmental variables.

### eDNA indices perform better than morphological ones

At stations within 50 m of the Armida platform, Ba and Zn in sediment reached concentrations at which adverse ecotoxicological effects become likely [47,72,73]. Pollution by Ba and Zn is commonly reported near gas production facilities [89,90] and is found in produced waters and marine sediments [90–92]. This potential pollution was reflected in the foraminiferal molecular indices. Notably, morphology-based indices, particularly Foram-AMBI, were less correlated with the distance from the platform and the concentrations of Ba and Zn. In turn, Foram-gAMBI was correlated with the distance from the platform, Ba, and Zn. This supports promising results on using foraminiferal metabarcoding in biomonitoring studies [45,46,52].

The global health of benthic habitats improves with increasing distance from the Armida platform, as assessed by EcoQS using foraminiferal molecular indices, but not by morphological indices, particularly Foram-AMBI. Indeed, the agreement between morphological and molecular foraminiferal indices is rather weak. Specifically, EcoQS obtained with gEQR and Foram-gAMBI better mirrored the pollution gradient induced by the Ba enrichment than those of EQR and Foram-AMBI (based on morphology), respectively. This may be explained by the higher number of MOTUs and species richness in the eDNA dataset compared to the number of morphospecies and diversity in the morphological one. Specifically, organic-walled foraminifera are very abundant in the eDNA assemblage, while they were not recorded in the morphology-based study, where benthic foraminifera were dried before picking hampering the preservation of organic-walled species. Dry-picking has the advantage of being less time-consuming than wet-picking, although the latter enables the preservation organic-walled foraminifera. Metabarcoding may contribute to capture organic-walled foraminifera diversity and ecology more easily than traditional morpho-taxonomy-based methods. However, it is worth mentioning that the incongruence between morphological and metabarcoding data can also be due to the differences in sampling methodology. By isolating eDNA from surface sediment (0–1 cm) we reduced the potential impact of the DNA preserved in the sediment for longer time, but at the same time our metabarcoding data do not include infaunal species that form part of the foraminiferal community.

The discrepancies observed between Foram-AMBI and Foram-gAMBI could also be ascribed to the different environmental parameter used for species and MOTUs assignment to EGs (i.e., TOC for the former and Ba for the latter). This result is in line with the limitations of Foram-AMBI to detect pollution gradient that are not of organic matter enrichment origin. As a matter of fact, the application of AMBI (with macrofauna) and Foram-AMBI (based on morphology) has achieved controversial results in the Mediterranean Sea [93,94]. For instance, AMBI did not perform well in detecting the degradation of benthic habitats in the lagoon of Venice in Italy [94], in Italian lagoons in the Adriatic Sea [93]. The same applies to Foram-AMBI in the Gulf of Gabès in Tunisia [31], in the Mar Piccolo in Italy [95] and in the Gulf of Manfredonia in the Adriatic Sea [96]. Furthermore, AMBI and Foram-AMBI do not constantly reflect well pollution by element trace metals like in the Northern Bohai Sea in China [97] and in Korea [98] for the former, and in the Gulf of Gabès [31] and in the Guanabara bay in Brazil [99].

Interestingly, there were also discrepancies among the molecular indices. Specifically, EcoQS depicted by Foram-gAMBI was slightly better than gEQR. This might be explained by the different concepts of these two indices: the former is based on the relative percentages of sensitive/indifferent/tolerant/opportunistic species, while the latter is based on a measure of entropy accounting for species dominance. It remained, however, difficult to observe a strong, harmful effect of the Armida platform. The EcoQS showed a poor/moderate status with gEQR and a moderate status in the vicinity of the platform station with Foram-gAMBI. This suggests a moderate impact of the platform on foraminiferal communities (i.e., MOTUs). Similar results have been previously reported for gas platforms in the Adriatic Sea and in the North Sea [42,90,91,100]. Monitoring the environmental effects of gas platforms is complex to quantify and interpret, as it is difficult to distinguish between naturally occurring abiotic and biotic parameters and pollution induced by the platform [90]. Notably, the Adriatic Sea’s physical features, i.e., its long, narrow topography, enhance the effects of salinity, temperature, abundant freshwater inputs of nutrients, oxygen concentration, and primary production [101] and hence may interact with the potential impacts of the platform.

Assessing EcoQS with indices calculated with eDNA data using boundaries defined with morphospecies may explain why we did not obtain a full agreement between gEQR and Foram-gAMBI. Intercalibration exercises may further help to adapt morphospecies-based criteria to eDNA-based indices [e.g., 26,81]. Defining reference conditions adapted to open environments in the Adriatic Sea for Foram-gAMBI may help improving its accuracy, since the assessment of EcoQS is based on the extent of deviation from reference conditions (WFD, 2000/60/EC). It is crucial to define the latter to obtain a valid classification. These improvements will facilitate the implementation of Foram-gAMBI.

## Conclusions

The present study tested the effectiveness of eDNA-based foraminiferal indices in assessing the impact of a gas platform in the Adriatic Sea. Our results confirm the potential of eDNA metabarcoding as a complement to morpho-taxonomy. Foraminiferal eDNA indices correlate well with the Ba as the main abiotic parameter. Using a taxonomy-free approach enables us to overcome the lack of ecological knowledge for most foraminiferal species in the eDNA datasets. This study provides evidence for higher sensitivity of eDNA-based foraminiferal indices compared to morphological ones. Because the ecological groups were calibrated against locally derived barium thresholds, their transferability to other geographic regions remains to be validated and should be tested in future studies. Furthermore, this study highlights the ecological importance of organic-walled monothalamous foraminifera, which may include key species that can serve as bioindicators for assessing offshore industrial activities. Furthermore, a massive effort must be pursed toward integrative taxonomy that involves a morphological and molecular (i.e., barcode) descriptions of species. This will lead to increase the assignment of MOTUs to morphospecies and facilitate the comparison of indices based on morphology and on eDNA metabarcoding. It will further contribute to improve our understanding of the functional biology of the detected taxa, for instance monothalamous species.

## Supporting information

S1 Fig Values (mean ± s.d.) of ass-Foram-gAMBI along the four transects (East, North, South and West) of the Armida gas platform. Corresponding EcoQS are reported (High: blue, Good: green, Moderate: yellow and Poor: orange).
(TIFF)

S2 Fig Correlation between ass-Foram-gAMBI and environmental parameters. Kendall’s coefficient of rank correlation (*τ*) is reported. Significant correlations are indicated: *: *p* < 0.05, **: *p* < 0.01, ***: *p* < 0.001.
(TIFF)

S1 Table Selected environmental parameters. (CSV)

S2 Table Morphological taxonomy data. Species raw counts and corresponding ecological groups for Foram-AMBI calculation.
(XLSX)

S3 Table MOTUs with corresponding morphospecies with corresponding ecological groups for ass-Foram-gAMBI calculation. (XLSX)

S4 Table Taxonomy-free procedure. MOTUs assignments to ecological groups and Foram-gAMBI calculation.
(XLSX)
