# Supplementary material for: Taxonomy-free approach overcomes the gaps in ecological knowledge: The case of foraminiferal metabarcoding applied to environmental impact assessment
Source: PLoS One. 2026 Aug 20;21(8):e0356357. doi: 10.1371/journal.pone.0356357 (PMC13492763; doi:10.1371/journal.pone.0356357)
Supplement: Supplementary file 2 [file pone.0356357.s009.docx]

Response letter

Dear editor

We thank Dr Marco Albano (reviewer#2) for his comments. We revised our ms according to his suggestions.

Reviewer #2: Dear Authors, thanks for following my previous suggestions on your manuscript. Looking at its revised draft, I found improvements from all points of view, especially for accuracy on methods and limitations.

We thank you for your comment.

Regarding my comment 12, and 1 of the other reviewers, I suggest improving your comments on the manuscript also in the discussion section utilising the same explanation that you add in the report to argue your choice:

"...the aim of data filtration, which is to remove the potential noise that could represent DNA molecules of extraneous origins (e.g. transported and preserved in the sediment). This DNA generally occurs at lower abundances and the common way to remove it is to filter out the low abundance sequence variants (ASVs). This also allows to remove the copies of rare genetic variants resulting from intragenomic polymorphism common in ribosomal operons of foraminifera. The filtration threshold is arbitrary. We choose 1000 reads to ensure that the analysed data correspond to the local population actually living there. We consider this step essential for the robustness of metabarcoding data in ecological studies."

I think it sounds clearer and more accurate to introduce the discussion of your results based on the applied methodology, and it could be useful for the readers.

We agree with the reviewer and revised the beginning of the discussion, which now reads, lines 463-480 : « *Environmental DNA-based biomonitoring with benthic foraminifera is sometimes challenged by the limitations of taxonomic reference databases and the limited ecological knowledge available for most metabarcoding data.* ***In addition, eDNA datasets can be affected by the presence of DNA molecules of extraneous origin (e.g., transported and preserved in the sediment), which may constitute background noise. To limit this, a common approach is to filter out low-abundance MOTUs, since this exogenous DNA generally occurs at lower abundances. This filtering step also removes rare genetic variants resulting from intragenomic polymorphism, which is common in the ribosomal operons of foraminifera. Although the filtration threshold is arbitrary, we chose 1000 reads to ensure that the analysed data corresponded to the local population actually living in situ. We consider this step essential for the robustness of metabarcoding data in ecological studies.***

*Beyond this filtering step, taxonomy-free metabarcoding offers additional advantages: by not assigning eDNA reads to specific taxonomic classes, this approach is not reliant on accurate taxonomic identification and is not limited by inadequate reference database coverage.* »

Same for the comment 8 response:

"...We decided to use part of the dataset for calibrating the assignment to ecological group to limit circular reasoning which could have severly biased our work. Different options could have been tested, like leave-one-station-out procedure. We considered that by working at replicate level, it allowed us to consider the full Ba gradient captured in this work, therefore providing a more robust calibration, hence assignment of MOTUs to eg for Foram-gAMBI calculation"

This could also be reported in the discussion section to clarify this aspect.

We followed the reviewer#2 comment and revised the discussion, lines 496-508: « *This study implements a taxonomy-free approach for EcoQS assessment with eDNA foraminiferal indices in the context of gas production in the Adriatic Sea. This approach allows us to produce a list of MOTUs and their corresponding ecological group assignments tailored to the observed pollution gradient (i.e., barium enrichment) typically observed near offshore gas platforms (60, 61).* ***The use of part of the dataset for calibrating the assignment to ecological group was mandatory to limit circular reasoning which could have severely biased our work. Alternative approaches could have been considered, such as a leave-one-station-out procedure (53) or machine-learning-based methods (57). We reasoned that working at the replicate level (i.e., leave-one-replicate-out) allowed us to capture the full Ba gradient represented in this dataset, rather than grouping replicates into discrete Ba concentration classes, which would have reduced the resolution of the gradient and introduced arbitrary class boundaries. This approach therefore provided a more robust calibration and, consequently, a more reliable assignment of MOTUs to ecological groups for Foram-gAMBI calculation.***
