# Supplementary material for: Taxonomy-free approach overcomes the gaps in ecological knowledge: The case of foraminiferal metabarcoding applied to environmental impact assessment
Source: PLoS One. 2026 Aug 20;21(8):e0356357. doi: 10.1371/journal.pone.0356357 (PMC13492763; doi:10.1371/journal.pone.0356357)
Supplement: Peer review history

# Peer review: Decision Letter 0

**DOI:** 10.1371/journal.pone.0356357.r001

**Authors:** Claudio D'Iglio

29 Apr 2026

PONE-D-26-15381

Taxonomy-free approach overcomes the gaps in ecological knowledge: the case of foraminiferal metabarcoding applied to environmental impact assessment

PLOS One

Dear Dr. Bouchet,

Thank you for submitting your manuscript to PLOS ONE. After careful consideration, we feel that it has merit but does not fully meet PLOS ONE’s publication criteria as it currently stands. Therefore, we invite you to submit a revised version of the manuscript that addresses the points raised during the review process.

Please follow the suggestions provided by reviewers to improve the sensitive points arisen during the revision process.

Please submit your revised manuscript by Jun 14 2026 11:59PM. If you will need more time than this to complete your revisions, please reply to this message or contact the journal office at plosone@plos.org. When you're ready to submit your revision, log on to https://www.editorialmanager.com/pone/ and select the 'Submissions Needing Revision' folder to locate your manuscript file.

Please include the following items when submitting your revised manuscript:

- A letter that responds to each point raised by the academic editor and reviewer(s). You should upload this letter as a separate file labeled 'Response to Reviewers'.
- A marked-up copy of your manuscript that highlights changes made to the original version. You should upload this as a separate file labeled 'Revised Manuscript with Track Changes'.
- An unmarked version of your revised paper without tracked changes. You should upload this as a separate file labeled 'Manuscript'.

If you would like to make changes to your financial disclosure, please include your updated statement in your cover letter. Guidelines for resubmitting your figure files are available below the reviewer comments at the end of this letter.

If applicable, we recommend that you deposit your laboratory protocols in protocols.io to enhance the reproducibility of your results. Protocols.io assigns your protocol its own identifier (DOI) so that it can be cited independently in the future. For instructions see: https://journals.plos.org/plosone/s/submission-guidelines#loc-laboratory-protocols. Additionally, PLOS ONE offers an option for publishing peer-reviewed Lab Protocol articles, which describe protocols hosted on protocols.io. Read more information on sharing protocols at https://plos.org/protocols?utm_medium=editorial-email&utm_source=authorletters&utm_campaign=protocols.

As the corresponding author, your ORCID iD is verified in the submission system and will appear in the published article. PLOS supports the use of ORCID, and we encourage all coauthors to register for an ORCID iD and use it as well. Please encourage your coauthors to verify their ORCID iD within the submission system before final acceptance, as unverified ORCID iDs will not appear in the published article. Only the individual author can complete the verification step; PLOS staff cannot verify ORCID iDs on behalf of authors.

We look forward to receiving your revised manuscript.

Kind regards,

Claudio D'Iglio, Ph.D.

Academic Editor

PLOS One

Journal Requirements:

When submitting your revision, we need you to address these additional requirements.

1. Please ensure that your manuscript meets PLOS ONE's style requirements, including those for file naming. The PLOS ONE style templates can be found at

https://journals.plos.org/plosone/s/file?id=wjVg/PLOSOne_formatting_sample_main_body.pdf and

https://journals.plos.org/plosone/s/file?id=ba62/PLOSOne_formatting_sample_title_authors_affiliations.pdf

2. Please note that PLOS One has specific guidelines on code sharing for submissions in which author-generated code underpins the findings in the manuscript. In these cases, we expect all author-generated code to be made available without restrictions upon publication of the work. Please review our guidelines at https://journals.plos.org/plosone/s/materials-and-software-sharing#loc-sharing-code and ensure that your code is shared in a way that follows best practice and facilitates reproducibility and reuse.

3. We noticed you have some minor occurrence of overlapping text with the following previous publication(s), which needs to be addressed:

- https://doi.org/10.1016/j.marenvres.2018.12.009

https://doi.org/10.1016/j.marenvres.2025.107608

In your revision ensure you cite all your sources (including your own works), and quote or rephrase any duplicated text outside the methods section. Further consideration is dependent on these concerns being addressed.

4. Thank you for stating the following in the Acknowledgments Section of your manuscript:

"The authors are very grateful to the SGS Italia staff for the sample collection. This study was partly supported by the Swiss National Science Foundation grants 31003A_179125 and4

316030_150817 (JP), and ENI spa, Linea di Business UpStream Distretto Centro-Settentrionale. Márcio S. dos S. de Jesus received a FAPESP grant for research internship abroad (Grant no. 2019/22902-2). Silvia H. M. Sousa is a Brazilian National Council for Scientific and Technological (CNPq) research fellow."

We note that you have provided additional information within the Acknowledgements Section that is not currently declared in your Funding Statement. Please note that funding information should not appear in the Acknowledgments section or other areas of your manuscript. We will only publish funding information present in the Funding Statement section of the online submission form.

Please remove any funding-related text from the manuscript and let us know how you would like to update your Funding Statement. Currently, your Funding Statement reads as follows:

"This study was partly supported by the Swiss National Science Foundation grants 31003A_179125 and

316030_150817 (JP), and ENI spa, Linea di Business UpStream Distretto Centro-Settentrionale. Márcio S. dos S. de Jesus received a FAPESP grant for research internship abroad (Grant no. 2019/22902-2)."

Please include your amended statements within your cover letter; we will change the online submission form on your behalf.

5. Thank you for stating the following financial disclosure:

"This study was partly supported by the Swiss National Science Foundation grants 31003A_179125 and

316030_150817 (JP), and ENI spa, Linea di Business UpStream Distretto Centro-Settentrionale. Márcio S. dos S. de Jesus received a FAPESP grant for research internship abroad (Grant no. 2019/22902-2)."

Please state what role the funders took in the study.  If the funders had no role, please state: "The funders had no role in study design, data collection and analysis, decision to publish, or preparation of the manuscript."

If this statement is not correct you must amend it as needed.

Please include this amended Role of Funder statement in your cover letter; we will change the online submission form on your behalf.

6. We noted in your submission details that a portion of your manuscript may have been presented or published elsewhere. "Part of the data were published in Cordier et al. (41) and Frontalini et al. (47). The current ms includes new unpublished data. In Cordier and Frontalini, the Foram-AMBI index was not used, the new ms provide new informations on the environmental quality, and is a very good study case to confirm the potential of taxonomy free procedure with eDNA data." Please clarify whether this [conference proceeding or publication] was peer-reviewed and formally published. If this work was previously peer-reviewed and published, in the cover letter please provide the reason that this work does not constitute dual publication and should be included in the current manuscript.

7. We note that Figure 1 in your submission contain [map/satellite] images which may be copyrighted. All PLOS content is published under the Creative Commons Attribution License (CC BY 4.0), which means that the manuscript, images, and Supporting Information files will be freely available online, and any third party is permitted to access, download, copy, distribute, and use these materials in any way, even commercially, with proper attribution. For these reasons, we cannot publish previously copyrighted maps or satellite images created using proprietary data, such as Google software (Google Maps, Street View, and Earth). For more information, see our copyright guidelines: http://journals.plos.org/plosone/s/licenses-and-copyright.

We require you to either (1) present written permission from the copyright holder to publish these figures specifically under the CC BY 4.0 license, or (2) remove the figures from your submission:

a. You may seek permission from the original copyright holder of Figure 1 to publish the content specifically under the CC BY 4.0 license.

We recommend that you contact the original copyright holder with the Content Permission Form (http://journals.plos.org/plosone/s/file?id=7c09/content-permission-form.pdf) and the following text:

“I request permission for the open-access journal PLOS ONE to publish XXX under the Creative Commons Attribution License (CCAL) CC BY 4.0 (http://creativecommons.org/licenses/by/4.0/). Please be aware that this license allows unrestricted use and distribution, even commercially, by third parties. Please reply and provide explicit written permission to publish XXX under a CC BY license and complete the attached form.”

Please upload the completed Content Permission Form or other proof of granted permissions as an ""Other"" file with your submission.

In the figure caption of the copyrighted figure, please include the following text: “Reprinted from [ref] under a CC BY license, with permission from [name of publisher], original copyright [original copyright year].”

b. If you are unable to obtain permission from the original copyright holder to publish these figures under the CC BY 4.0 license or if the copyright holder’s requirements are incompatible with the CC BY 4.0 license, please either i) remove the figure or ii) supply a replacement figure that complies with the CC BY 4.0 license. Please check copyright information on all replacement figures and update the figure caption with source information. If applicable, please specify in the figure caption text when a figure is similar but not identical to the original image and is therefore for illustrative purposes only.

The following resources for replacing copyrighted map figures may be helpful:

USGS National Map Viewer (public domain): http://viewer.nationalmap.gov/viewer/

The Gateway to Astronaut Photography of Earth (public domain): http://eol.jsc.nasa.gov/sseop/clickmap/

Maps at the CIA (public domain): https://www.cia.gov/library/publications/the-world-factbook/index.html and https://www.cia.gov/library/publications/cia-maps-publications/index.html

NASA Earth Observatory (public domain): http://earthobservatory.nasa.gov/

Landsat: http://landsat.visibleearth.nasa.gov/

USGS EROS (Earth Resources Observatory and Science (EROS) Center) (public domain): http://eros.usgs.gov/#

Natural Earth (public domain): http://www.naturalearthdata.com/

8. If the reviewer comments include a recommendation to cite specific previously published works, please review and evaluate these publications to determine whether they are relevant and should be cited. There is no requirement to cite these works unless the editor has indicated otherwise.

[Note: HTML markup is below. Please do not edit.]

Reviewers' comments:

Reviewer's Responses to Questions

**Comments to the Author**

1. Is the manuscript technically sound, and do the data support the conclusions?

Reviewer #1: Partly

Reviewer #2: Partly

**********

2. Has the statistical analysis been performed appropriately and rigorously?

Reviewer #1: N/A

Reviewer #2: Yes

**********

3. Have the authors made all data underlying the findings in their manuscript fully available?

The PLOS Data policy

Reviewer #1: Yes

Reviewer #2: No

**********

4. Is the manuscript presented in an intelligible fashion and written in standard English?

Reviewer #1: Yes

Reviewer #2: Yes

**********

Reviewer #1: The manuscript addresses an important and timely topic, namely the use of a taxonomy-free approach in foraminiferal eDNA metabarcoding for the assessment of the ecological quality of benthic habitats. The central idea is interesting and potentially valuable: the authors demonstrate that a taxonomy-free framework may exploit the molecular signal more effectively than conventional morphological or taxonomically assigned molecular indices, particularly in the presence of a high proportion of monothalamous taxa whose ecology remains poorly understood. The study is based on data collected around the Armida gas platform in the northern Adriatic Sea.

It is evident, and on this point I fully agree with the authors, that relying exclusively on morphologically identified species for ecological status assessment is insufficient. In my view, such an approach often fails to make full use of the available biodiversity data, which may in turn lead to questionable interpretations of the results.

In the Methods section, I would recommend explaining in greater detail why the SWARM clustering algorithm was selected and how exactly it was parameterized, so that it is clear how the MOTUs were defined. At line 211, it would also be appropriate to describe the filtering of HTS data, as this abbreviation has not been introduced previously. I also find the wording in lines 211–212 problematic: “to improve the congruence in terms of alpha diversity the MOTUs represented by less than 1000 reads were excluded.” This justification appears rather subjective. It is not clear what the reader should understand by “improve the congruence in terms of alpha diversity,” nor is it sufficiently explained why the threshold of 1000 reads was chosen. If I understand correctly, the algorithm originally produced nearly 23,000 MOTUs, but after excluding units represented by fewer than 1000 reads, only 343 remained. This represents a very substantial reduction of the dataset, exceeding 85%, and therefore the rationale for this step should be explained much more convincingly and in greater detail.

A similarly unclear step is presented at line 216: “The MOTUs assigned to the same morphospecies have been combined, reducing the total number of assigned MOTUs to 43.” If MOTUs were generated using the SWARM algorithm, and if the text simultaneously suggests that morphospecies may not reliably reflect actual diversity, then it is not entirely clear why MOTUs assigned to the same morphological species were subsequently merged. In my opinion, this procedure deserves a much clearer justification. Overall, I believe that the section between lines 206–217 is not formulated with sufficient clarity and in places appears overly subjective. I would recommend that this section be carefully revised, clarified, and reworked, because the resulting dataset is fundamental to all subsequent analyses and interpretations. It would also be useful to provide the results of the same analyses without these subjective dataset modifications, at least in the supplementary materials, so that the reader can assess whether, and to what extent, these steps affected the results. On the one hand, the authors emphasize the strength of DNA-based data; on the other hand, they substantially reduce that signal themselves.

The reference conditions for gEQR are defined internally from the 95th percentile of the investigated stations. This may be a practical solution, but if the entire study area already includes anthropogenically affected stations, the “reference conditions” may not be truly reference conditions. As a result, the classification of gEQR may be biased. The authors should state this limitation more explicitly in both the Methods and the Discussion and explain why this procedure is acceptable in the present context. The use of the 95th percentile as the reference value appears rather risky to me, and in my opinion it would be appropriate to explain whether no alternative method of deriving reference values was available, or why such an alternative was not used.

In presenting the results, I would recommend harmonizing the orientation of the scales in Figure 5. At present, EQR and gEQR are displayed with EcoQS running from “High” at the top to “Bad” at the bottom, whereas AMBI and gAMBI are oriented in the opposite direction, i.e. from “Moderate” at the top to “High” at the bottom. I understand that this reflects the values of the indices themselves, as one increases while the other decreases, but in the figures the EcoQS categories are more important than the raw index values. The current arrangement may therefore be confusing for the reader, and a unified orientation would be preferable.

At lines 267–268, the meaning of the statement “Replicates were partitioned by identity (A, B, and C), and in each iteration” is not sufficiently clear to me. The Methods indicate that three replicates were collected from each core, but later in the DNA methodology it is stated that three replicates were generated from each sample. I therefore cannot deduce unambiguously whether three replicates per sampling site were sequenced, or whether each biological replicate was further processed into three PCR replicates, resulting in a total of nine sequenced replicates. Likewise, it is not clear what exactly the A, B, and C partitions represent. This part of the methodology should therefore be specified much more precisely. At the same time, it is not explained why the authors chose this particular partitioning scheme for the leave-one-replicate-out procedure. For example, it is unclear why the replicates were not grouped according to classes of Ba concentration, given that this variable plays a central role in the study.

I will not assess the statistical analyses in detail, as this is not among my main areas of expertise. Nevertheless, I would like to comment on the interpretation of some of the results. At line 355, a trend in Exp(H) is reported; however, in my opinion, Figure 3 does not show a clear trend either in Exp(H) or in DNA-based diversity. I do not know whether such a trend could be formally tested, but based on the figures alone I would be much more cautious in making statements about trends.

More generally, I have the impression that the results based on EQR and AMBI are at times interpreted more in line with the authors’ expectations than strictly according to how they are presented in the figures. For example, lines 399–400 state: “Conversely, EcoQS worsened with Foram-AMBI along the 0 to 2000 m transects,” yet this conclusion does not seem unambiguous to me from the corresponding graphs. In my opinion, the East transect does not show a deterioration in EcoQS, and neither does the West transect, apart from a minor deviation around 50 m. I perceive a similar mismatch between the text and the visually presented results in other parts of Figure 5 as well. I would therefore recommend reconsidering the interpretation of the results in this section and formulating it more conservatively, so that it more faithfully reflects the graphical outputs themselves.

In conclusion, I regard the taxonomy-free approach to DNA metabarcoding data as very promising and, given the current limitations of classical morphotaxonomy and the exponentially increasing volume of new data, essentially the only realistic and appropriate way forward. The manuscript has several strengths: a clearly articulated motivation, a relevant applied research question, and a convincing argument that a substantial portion of the biological signal in eDNA datasets remains unused when the analysis relies solely on taxonomically assigned MOTUs. However, the reported results and their interpretation do not yet appear fully convincing to me.

Reviewer #2: The paper's draft "Taxonomy-free approach overcomes the gaps in ecological knowledge: the case of foraminiferal metabarcoding applied to environmental impact assessment" of Bouchet and colleagues deals with a critical challenge in marine biomonitoring: the inability of traditional (morphology-based) methods to capture the full diversity of foraminifera, particularly soft-walled taxa (monothalamids), which are often dominant but difficult to identify. The document proposes and validates a taxonomy-free approach based on environmental DNA (eDNA) metabarcoding, using the "Armida" gas platform in the Adriatic Sea as a case study. Once published, this article could represent a high-value contribution that shifts the biomonitoring paradigm towards genomic ecology. It successfully demonstrates that it is not necessary to know the "name" of each organism to assess the health of an ecosystem, as long as one knows its response to environmental stress. It is a pioneering work that facilitates the adoption of eDNA in international regulations (such as the European WFD). The value of this manuscript is potentially high, but it needs some revision in this sense.

Both the title and abstract are effective, designed to maximise the visibility and key points of the contribution. I suggest removing the keywords already reported in the title and replacing them with related ones to improve the document's soundness. Probably adding some numeric key results in the abstract (eg, eDNA/morphology MOTUs) could enhance the soundness of your data.

The introduction section is well-drafted with all the necessary information given to the reader to understand the methods and the author's research question.

The sampling design looks properly set, with adequate numbers and replicas, scientifically strong, even if some confusion is generating later in their "partitioning", what do you mean with replicas partitioning?

Methods and analyses were well applied and supported by previous literature, also from the authors wich demonstrate experience in this regard between the lines. Rather, I have some doubts about the data filtration with a threshold of 1000 reads. Due to the nature of the sampled matrix and the specific biomarker, could it represent a correct choice, or was it influenced by some methodological limitations? Please argue this better.

A point to better argue in the discussion is that eDNA can persist in the sediment for a long time, even after the organisms die. Although the authors cite the preservation kit, a more in-depth discussion of the distinction between "live" DNA and "dead" DNA could add more accuracy to the study results, even to contextualise whether the indices reflect the platform's current health or an average over recent years. The authors used the 0-1 cm layer to mitigate this problem, which is the correct standard procedure, but at the same time, the influence of this problem also affects the methodology (short length MOTUs), which could be discussed and integrated. Moreover, I suggest depositing all the raw data in a public repository to give the reader access.

The limitations of the approach were adequately stated by thre authors, such as the influence of river of the study area on the chemical equilibria. The link between barium and gas platforms is supported by a solid literature, confirming that the choice of stress parameter is correct. Since the approach is "calibrated" to local Barium, the transferability of the ecological groups defined in this study to other geographical areas requires further validation. Since the Barium thresholds (Table 1) were created by the authors themselves because they did not exist in the literature, this point remain a potential limitations of this study replication by other authors, to hihglight in the conclusion section.

Although, as specified by the authors, the lack of ecological knowledge on monothalamids is a bottleneck that only the taxonomy-free approach can currently circumvent. Indeed, even if the approach of this study is taxonomy-free, improving genomic databases remains essential to better understand the functional biology of the detected taxa, which could be well-assessed in the conclusion section too, which in the present form resulted poor in this sense, focusing just on the effectivenes of the method/biomarker proposed (proper results).

Best regards

**********

what does this mean?). If published, this will include your full peer review and any attached files.

If you choose “no”, your identity will remain anonymous but your review may still be made public.

**Do you want your identity to be public for this peer review?**  For information about this choice, including consent withdrawal, please see our Privacy Policy

Reviewer #1: No

Reviewer #2: No

**********

[NOTE: If reviewer comments were submitted as an attachment file, they will be attached to this email and accessible via the submission site. Please log into your account, locate the manuscript record, and check for the action link "View Attachments". If this link does not appear, there are no attachment files.]

To ensure your figures meet our technical requirements, please review our figure guidelines: https://journals.plos.org/plosone/s/figures

You may also use PLOS’s free figure tool, NAAS, to help you prepare publication quality figures: https://journals.plos.org/plosone/s/figures#loc-tools-for-figure-preparation.

NAAS will assess whether your figures meet our technical requirements by comparing each figure against our figure specifications.

# Author comment: Author response to Decision Letter 1

**DOI:** 10.1371/journal.pone.0356357.r002

14 May 2026

Journal Requirements:

When submitting your revision, we need you to address these additional requirements.

1. Please ensure that your manuscript meets PLOS ONE's style requirements, including those for file naming. The PLOS ONE style templates can be found at

https://journals.plos.org/plosone/s/file?id=wjVg/PLOSOne_formatting_sample_main_body.pdf and

https://journals.plos.org/plosone/s/file?id=ba62/PLOSOne_formatting_sample_title_authors_affiliations.pdf

We edited our ms to meet PLOS ONE’s style requirements.

2. Please note that PLOS One has specific guidelines on code sharing for submissions in which author-generated code underpins the findings in the manuscript. In these cases, we expect all author-generated code to be made available without restrictions upon publication of the work. Please review our guidelines at https://journals.plos.org/plosone/s/materials-and-software-sharing#loc-sharing-code and ensure that your code is shared in a way that follows best practice and facilitates reproducibility and reuse.

3. We noticed you have some minor occurrence of overlapping text with the following previous publication(s), which needs to be addressed:

- https://doi.org/10.1016/j.marenvres.2018.12.009

https://doi.org/10.1016/j.marenvres.2025.107608

In your revision ensure you cite all your sources (including your own works), and quote or rephrase any duplicated text outside the methods section. Further consideration is dependent on these concerns being addressed.

We are surprised by this comment. Outside the method section, the « Compilatio » platform (online access provide by the University of Lille) hardly detected any overlapping text with some of our previous publications and other publications.

We would be happy to proceed with quoting/rephrasing if you could provide us with the overlapping text you detected.

4. Thank you for stating the following in the Acknowledgments Section of your manuscript:

"The authors are very grateful to the SGS Italia staff for the sample collection. This study was partly supported by the Swiss National Science Foundation grants 31003A_179125 and 316030_150817 (JP), and ENI spa, Linea di Business UpStream Distretto Centro-Settentrionale. Márcio S. dos S. de Jesus received a FAPESP grant for research internship abroad (Grant no. 2019/22902-2). Silvia H. M. Sousa is a Brazilian National Council for Scientific and Technological (CNPq) research fellow."

We note that you have provided additional information within the Acknowledgements Section that is not currently declared in your Funding Statement. Please note that funding information should not appear in the Acknowledgments section or other areas of your manuscript. We will only publish funding information present in the Funding Statement section of the online submission form.

Please remove any funding-related text from the manuscript and let us know how you would like to update your Funding Statement. Currently, your Funding Statement reads as follows:

"This study was partly supported by the Swiss National Science Foundation grants 31003A_179125 and 316030_150817 (JP), and ENI spa, Linea di Business UpStream Distretto Centro-Settentrionale. Márcio S. dos S. de Jesus received a FAPESP grant for research internship abroad (Grant no. 2019/22902-2)."

Please include your amended statements within your cover letter; we will change the online submission form on your behalf.

There is no need to change our funding statement, and we modified the acknowledgements section accordingly.

5. Thank you for stating the following financial disclosure:

"This study was partly supported by the Swiss National Science Foundation grants 31003A_179125 and

316030_150817 (JP), and ENI spa, Linea di Business UpStream Distretto Centro-Settentrionale. Márcio S. dos S. de Jesus received a FAPESP grant for research internship abroad (Grant no. 2019/22902-2)."

Please state what role the funders took in the study. If the funders had no role, please state: "The funders had no role in study design, data collection and analysis, decision to publish, or preparation of the manuscript."

If this statement is not correct you must amend it as needed.

Please include this amended Role of Funder statement in your cover letter; we will change the online submission form on your behalf.

The statement is correct “"The funders had no role in study design, data collection and analysis, decision to publish, or preparation of the manuscript."”

6. We noted in your submission details that a portion of your manuscript may have been presented or published elsewhere. "Part of the data were published in Cordier et al. (41) and Frontalini et al. (47). The current ms includes new unpublished data. In Cordier and Frontalini, the Foram-AMBI index was not used, the new ms provide new informations on the environmental quality, and is a very good study case to confirm the potential of taxonomy free procedure with eDNA data." Please clarify whether this [conference proceeding or publication] was peer-reviewed and formally published. If this work was previously peer-reviewed and published, in the cover letter please provide the reason that this work does not constitute dual publication and should be included in the current manuscript.

We responded to this comment in the cover letter. The work was previously published in peer-reviewed journal i.e. Cordier et al. (41) and Frontalini et al. (47). As explained, the current ms add new data on the morphological dataset, the Foram-AMBI index was not calculated in Cordier et al. (41) and Frontalini et al. (47) as only latest development now allow us to compute it, the new ms hence provide new informations on the environmental quality, and is a very good study case to confirm the potential of taxonomy free procedure with eDNA data. It does not therefore consitutute a dual publication.

7. We note that Figure 1 in your submission contain [map/satellite] images which may be copyrighted. All PLOS content is published under the Creative Commons Attribution License (CC BY 4.0), which means that the manuscript, images, and Supporting Information files will be freely available online, and any third party is permitted to access, download, copy, distribute, and use these materials in any way, even commercially, with proper attribution. For these reasons, we cannot publish previously copyrighted maps or satellite images created using proprietary data, such as Google software (Google Maps, Street View, and Earth). For more information, see our copyright guidelines: http://journals.plos.org/plosone/s/licenses-and-copyright.

We require you to either (1) present written permission from the copyright holder to publish these figures specifically under the CC BY 4.0 license, or (2) remove the figures from your submission:

a. You may seek permission from the original copyright holder of Figure 1 to publish the content specifically under the CC BY 4.0 license.

We recommend that you contact the original copyright holder with the Content Permission Form (http://journals.plos.org/plosone/s/file?id=7c09/content-permission-form.pdf) and the following text:

“I request permission for the open-access journal PLOS ONE to publish XXX under the Creative Commons Attribution License (CCAL) CC BY 4.0 (http://creativecommons.org/licenses/by/4.0/). Please be aware that this license allows unrestricted use and distribution, even commercially, by third parties. Please reply and provide explicit written permission to publish XXX under a CC BY license and complete the attached form.”

Please upload the completed Content Permission Form or other proof of granted permissions as an ""Other"" file with your submission.

In the figure caption of the copyrighted figure, please include the following text: “Reprinted from [ref] under a CC BY license, with permission from [name of publisher], original copyright [original copyright year].”

b. If you are unable to obtain permission from the original copyright holder to publish these figures under the CC BY 4.0 license or if the copyright holder’s requirements are incompatible with the CC BY 4.0 license, please either i) remove the figure or ii) supply a replacement figure that complies with the CC BY 4.0 license. Please check copyright information on all replacement figures and update the figure caption with source information. If applicable, please specify in the figure caption text when a figure is similar but not identical to the original image and is therefore for illustrative purposes only.

The following resources for replacing copyrighted map figures may be helpful:

USGS National Map Viewer (public domain): http://viewer.nationalmap.gov/viewer/

The Gateway to Astronaut Photography of Earth (public domain): http://eol.jsc.nasa.gov/sseop/clickmap/

Maps at the CIA (public domain): https://www.cia.gov/library/publications/the-world-factbook/index.html and https://www.cia.gov/library/publications/cia-maps-publications/index.html

NASA Earth Observatory (public domain): http://earthobservatory.nasa.gov/

Landsat: http://landsat.visibleearth.nasa.gov/

USGS EROS (Earth Resources Observatory and Science (EROS) Center) (public domain): http://eros.usgs.gov/#

Natural Earth (public domain): http://www.naturalearthdata.com/

The figure was remade using R software packages ‘rnaturalearth’ and ‘rnaturalearthdata’. These package are using raster and vector map from Natural Earth website, the data coming from this website being in the public domain. As indicated on the Natural Earth website «No permission is needed to use Natural Earth. Crediting the authors is unnecessary ». Therefore there is no more copyright issue.

Reviewer #1:

The manuscript addresses an important and timely topic, namely the use of a taxonomy-free approach in foraminiferal eDNA metabarcoding for the assessment of the ecological quality of benthic habitats. The central idea is interesting and potentially valuable: the authors demonstrate that a taxonomy-free framework may exploit the molecular signal more effectively than conventional morphological or taxonomically assigned molecular indices, particularly in the presence of a high proportion of monothalamous taxa whose ecology remains poorly understood. The study is based on data collected around the Armida gas platform in the northern Adriatic Sea.

It is evident, and on this point I fully agree with the authors, that relying exclusively on morphologically identified species for ecological status assessment is insufficient. In my view, such an approach often fails to make full use of the available biodiversity data, which may in turn lead to questionable interpretations of the results.

We thank the reviewer #1 for these comments.

Comment n°1: In the Methods section, I would recommend explaining in greater detail why the SWARM clustering algorithm was selected and how exactly it was parameterized, so that it is clear how the MOTUs were defined. At line 211, it would also be appropriate to describe the filtering of HTS data, as this abbreviation has not been introduced previously. I also find the wording in lines 211–212 problematic: “to improve the congruence in terms of alpha diversity the MOTUs represented by less than 1000 reads were excluded.” This justification appears rather subjective. It is not clear what the reader should understand by “improve the congruence in terms of alpha diversity,” nor is it sufficiently explained why the threshold of 1000 reads was chosen. If I understand correctly, the algorithm originally produced nearly 23,000 MOTUs, but after excluding units represented by fewer than 1000 reads, only 343 remained. This represents a very substantial reduction of the dataset, exceeding 85%, and therefore the rationale for this step should be explained much more convincingly and in greater detail.

Response n°1: The SWARM v2.1.8 algorithm was used with the default d parameter (i.e., 1), as specified in the Frontalini et al. 2020 paper. This information is now added to our paper.

Regarding the data filtration issue, the sentence was modified as follows:

“To remove the noise in our data due to the presence of rare genetic variants or eDNA molecules transported from other areas, which could influence the alpha diversity analysis, we filtered out the MOTUs represented by less than 1000 reads.”

We hope that this additional statement helps explaining the aim of data filtration, which is to remove the potential noise that could represent DNA molecules of extraneous origins (e.g. transported and preserved in the sediment). This DNA generally occurs at lower abundances and the common way to remove it is to filter out the low abundance sequence variants (ASVs). This also allows to remove the copies of rare genetic variants resulting from intragenomic polymorphism common in ribosomal operons of foraminifera. The filtration threshold is arbitrary. We choose 1000 reads to ensure that the analysed data correspond to the local population actually living there. We consider this step essential for the robustness of metabarcoding data in ecological studies.

Comment n°2: A similarly unclear step is presented at line 216: “The MOTUs assigned to the same morphospecies have been combined, reducing the total number of assigned MOTUs to 43.” If MOTUs were generated using the SWARM algorithm, and if the text simultaneously suggests that morphospecies may not reliably reflect actual diversity, then it is not entirely clear why MOTUs assigned to the same morphological species were subsequently merged. In my opinion, this procedure deserves a much clearer justification.

Response n°2: The MOTUs assigned to the same morphospecies were combined to facilitate the comparison of molecular and morphological data. Indeed, each morphospecies is composed of a number of genetically distinctive MOTUs. Here, we assumed that the MOTUs assigned to the same morphospecies are characterized by the same ecology.

We added a statement to explain the reasons why the MOTUs were combined.

Comment n°3 : Overall, I believe that the section between lines 206–217 is not formulated with sufficient clarity and in places appears overly subjective. I would recommend that this section be carefully revised, clarified, and reworked, because the resulting dataset is fundamental to all subsequent analyses and interpretations. It would also be useful to provide the results of the same analyses without these subjective dataset modifications, at least in the supplementary materials, so that the reader can assess whether, and to what extent, these steps affected the results. On the one hand, the authors emphasize the strength of DNA-based data; on the other hand, they substantially reduce that signal themselves.

Response n°3: We are now providing more in-depth explanations for our methodological framework for the eDNA data (see response n°1, n°2, n°12 and n°13).

Comment n°4: The reference conditions for gEQR are defined internally from the 95th percentile of the investigated stations. This may be a practical solution, but if the entire study area already includes anthropogenically affected stations, the “reference conditions” may not be truly reference conditions. As a result, the classification of gEQR may be biased. The authors should state this limitation more explicitly in both the Methods and the Discussion and explain why this procedure is acceptable in the present context. The use of the 95th percentile as the reference value appears rather risky to me, and in my opinion it would be appropriate to explain whether no alternative method of deriving reference values was available, or why such an alternative was not used.

Response n°4: The Ba and Zn concentrations (Table S4) are fairly low at the stations farther away from the platforms as shown by the PCA. The PCA (Fig. 2) further highlights the difference between the polluted and the unpolluted stations. We hence think that using the 95th percentile is a conservative approach, further supported by the fact that some stations are unpolluted, to determine truly reference conditions to obtain an unbiased gEQR.

The methods section now reads: «Local-specific reference conditions are the anchor points for calculatin

Attachment Submitted filename: response_to_reviewers.docx

# Peer review: Decision Letter 1

**DOI:** 10.1371/journal.pone.0356357.r003

**Authors:** Claudio D'Iglio

27 Jul 2026

Dear Dr. Bouchet,

Thank you for submitting your manuscript to PLOS ONE. After careful consideration, we feel that it has merit but does not fully meet PLOS ONE’s publication criteria as it currently stands. Therefore, we invite you to submit a revised version of the manuscript that addresses the points raised during the review process.

Please submit your revised manuscript by Sep 10 2026 11:59PM. If you will need more time than this to complete your revisions, please reply to this message or contact the journal office at plosone@plos.org. When you're ready to submit your revision, log on to https://www.editorialmanager.com/pone/ and select the 'Submissions Needing Revision' folder to locate your manuscript file.

- A letter that responds to each point raised by the academic editor and reviewer(s). You should upload this letter as a separate file labeled 'Response to Reviewers'.
- A marked-up copy of your manuscript that highlights changes made to the original version. You should upload this as a separate file labeled 'Revised Manuscript with Track Changes'.
- An unmarked version of your revised paper without tracked changes. You should upload this as a separate file labeled 'Manuscript'.

If you would like to make changes to your financial disclosure, please include your updated statement in your cover letter. Guidelines for resubmitting your figure files are available below the reviewer comments at the end of this letter.

If applicable, we recommend that you deposit your laboratory protocols in protocols.io to enhance the reproducibility of your results. Protocols.io assigns your protocol its own identifier (DOI) so that it can be cited independently in the future. For instructions see: https://journals.plos.org/plosone/s/submission-guidelines#loc-laboratory-protocols. Additionally, PLOS ONE offers an option for publishing peer-reviewed Lab Protocol articles, which describe protocols hosted on protocols.io. Read more information on sharing protocols at https://plos.org/protocols?utm_medium=editorial-email&utm_source=authorletters&utm_campaign=protocols.

As the corresponding author, your ORCID iD is verified in the submission system and will appear in the published article. PLOS supports the use of ORCID, and we encourage all coauthors to register for an ORCID iD and use it as well. Please encourage your coauthors to verify their ORCID iD within the submission system before final acceptance, as unverified ORCID iDs will not appear in the published article. *Only*  the individual author can complete the verification step; PLOS staff *cannot*  verify ORCID iDs on behalf of authors.

We look forward to receiving your revised manuscript.

Kind regards,

Claudio D'Iglio, Ph.D.

Academic Editor

PLOS One

Journal Requirements:

If the reviewer comments include a recommendation to cite specific previously published works, please review and evaluate these publications to determine whether they are relevant and should be cited. There is no requirement to cite these works unless the editor has indicated otherwise.

Please review your reference list to ensure that it is complete and correct. If you have cited papers that have been retracted, please include the rationale for doing so in the manuscript text, or remove these references and replace them with relevant current references. Any changes to the reference list should be mentioned in the rebuttal letter that accompanies your revised manuscript. If you need to cite a retracted article, indicate the article’s retracted status in the References list and also include a citation and full reference for the retraction notice.

**Additional Editor Comments:**

Please improve some point of the Discussion section to resolve the minor concerns that emerged from revison. Please follow the reviewer suggestions.

[Note: HTML markup is below. Please do not edit.]

Reviewers' comments:

Reviewer's Responses to Questions

**Comments to the Author**

Reviewer #2: (No Response)

Reviewer #3: All comments have been addressed

**********

2. Is the manuscript technically sound, and do the data support the conclusions?

Reviewer #2: Yes

Reviewer #3: Yes

**********

3. Has the statistical analysis been performed appropriately and rigorously?

Reviewer #2: Yes

Reviewer #3: Yes

**********

4. Have the authors made all data underlying the findings in their manuscript fully available?

The PLOS Data policy

Reviewer #2: Yes

Reviewer #3: Yes

**********

5. Is the manuscript presented in an intelligible fashion and written in standard English?

Reviewer #2: Yes

Reviewer #3: Yes

**********

Reviewer #2: Dear Authors, thanks for following my previous suggestions on your manuscript. Looking at its revised draft, I found improvements from all points of view, especially for accuracy on methods and limitations.

Regarding my comment 12, and 1 of the other reviewers, I suggest improving your comments on the manuscript also in the discussion section utilising the same explanation that you add in the report to argue your choice:

"...the aim of data filtration, which is to remove the potential noise that could represent DNA molecules of extraneous origins (e.g. transported and preserved in the sediment). This DNA generally occurs at lower abundances and the common way to remove it is to filter out the low abundance sequence variants (ASVs). This also allows to remove the copies of rare genetic variants resulting from intragenomic polymorphism common in ribosomal operons of foraminifera. The filtration threshold is arbitrary. We choose 1000 reads to ensure that the analysed data correspond to the local population actually living there. We consider this step essential for the robustness of metabarcoding data in ecological studies."

I think it sounds clearer and more accurate to introduce the discussion of your results based on the applied methodology, and it could be useful for the readers.

Same for the comment 8 response:

"...We decided to use part of the dataset for calibrating the assignment to ecological group to limit circular reasoning which could have severly biased our work. Different options could have been tested, like leave-one-station-out procedure. We considered that by working at replicate level, it allowed us to consider the full Ba gradient captured in this work, therefore providing a more robust calibration, hence assignment of MOTUs to eg for Foram-gAMBI calculation"

This could also be reported in the discussion section to clarify this aspect.

Best regards

The Reviewer

Reviewer #3: (No Response)

**********

what does this mean?). If published, this will include your full peer review and any attached files.

If you choose “no”, your identity will remain anonymous but your review may still be made public.

**Do you want your identity to be public for this peer review?**  For information about this choice, including consent withdrawal, please see our Privacy Policy

Reviewer #2: **Yes:** Marco Albano

Reviewer #3: **Yes:** LAZARO LUIZ MATTOS LAUT

**********

[NOTE: If reviewer comments were submitted as an attachment file, they will be attached to this email and accessible via the submission site. Please log into your account, locate the manuscript record, and check for the action link "View Attachments". If this link does not appear, there are no attachment files.]

To ensure your figures meet our technical requirements, please review our figure guidelines: https://journals.plos.org/plosone/s/figures

You may also use PLOS’s free figure tool, NAAS, to help you prepare publication quality figures: https://journals.plos.org/plosone/s/figures#loc-tools-for-figure-preparation.

NAAS will assess whether your figures meet our technical requirements by comparing each figure against our figure specifications.

# Author comment: Author response to Decision Letter 2

**DOI:** 10.1371/journal.pone.0356357.r004

28 Jul 2026

Response letter

Dear editor

We thank Dr Marco Albano (reviewer#2) for his comments. We revised our ms according to his suggestions.

Reviewer #2: Dear Authors, thanks for following my previous suggestions on your manuscript. Looking at its revised draft, I found improvements from all points of view, especially for accuracy on methods and limitations.

We thank you for your comment.

Regarding my comment 12, and 1 of the other reviewers, I suggest improving your comments on the manuscript also in the discussion section utilising the same explanation that you add in the report to argue your choice:

"...the aim of data filtration, which is to remove the potential noise that could represent DNA molecules of extraneous origins (e.g. transported and preserved in the sediment). This DNA generally occurs at lower abundances and the common way to remove it is to filter out the low abundance sequence variants (ASVs). This also allows to remove the copies of rare genetic variants resulting from intragenomic polymorphism common in ribosomal operons of foraminifera. The filtration threshold is arbitrary. We choose 1000 reads to ensure that the analysed data correspond to the local population actually living there. We consider this step essential for the robustness of metabarcoding data in ecological studies."

I think it sounds clearer and more accurate to introduce the discussion of your results based on the applied methodology, and it could be useful for the readers.

We agree with the reviewer and revised the beginning of the discussion, which now reads, lines 463-480 : « Environmental DNA-based biomonitoring with benthic foraminifera is sometimes challenged by the limitations of taxonomic reference databases and the limited ecological knowledge available for most metabarcoding data. In addition, eDNA datasets can be affected by the presence of DNA molecules of extraneous origin (e.g., transported and preserved in the sediment), which may constitute background noise. To limit this, a common approach is to filter out low-abundance MOTUs, since this exogenous DNA generally occurs at lower abundances. This filtering step also removes rare genetic variants resulting from intragenomic polymorphism, which is common in the ribosomal operons of foraminifera. Although the filtration threshold is arbitrary, we chose 1000 reads to ensure that the analysed data corresponded to the local population actually living in situ. We consider this step essential for the robustness of metabarcoding data in ecological studies.

Beyond this filtering step, taxonomy-free metabarcoding offers additional advantages: by not assigning eDNA reads to specific taxonomic classes, this approach is not reliant on accurate taxonomic identification and is not limited by inadequate reference database coverage. »

Same for the comment 8 response:

"...We decided to use part of the dataset for calibrating the assignment to ecological group to limit circular reasoning which could have severly biased our work. Different options could have been tested, like leave-one-station-out procedure. We considered that by working at replicate level, it allowed us to consider the full Ba gradient captured in this work, therefore providing a more robust calibration, hence assignment of MOTUs to eg for Foram-gAMBI calculation"

This could also be reported in the discussion section to clarify this aspect.

We followed the reviewer#2 comment and revised the discussion, lines 496-508: « This study implements a taxonomy-free approach for EcoQS assessment with eDNA foraminiferal indices in the context of gas production in the Adriatic Sea. This approach allows us to produce a list of MOTUs and their corresponding ecological group assignments tailored to the observed pollution gradient (i.e., barium enrichment) typically observed near offshore gas platforms (60, 61). The use of part of the dataset for calibrating the assignment to ecological group was mandatory to limit circular reasoning which could have severely biased our work. Alternative approaches could have been considered, such as a leave-one-station-out procedure (53) or machine-learning-based methods (57). We reasoned that working at the replicate level (i.e., leave-one-replicate-out) allowed us to capture the full Ba gradient represented in this dataset, rather than grouping replicates into discrete Ba concentration classes, which would have reduced the resolution of the gradient and introduced arbitrary class boundaries. This approach therefore provided a more robust calibration and, consequently, a more reliable assignment of MOTUs to ecological groups for Foram-gAMBI calculation.

Attachment Submitted filename: response_to_reviewers_auresp_2.docx

# Editor report: Decision Letter 2

**DOI:** 10.1371/journal.pone.0356357.r005

**Authors:** Claudio D'Iglio

3 Aug 2026

Taxonomy-free approach overcomes the gaps in ecological knowledge: the case of foraminiferal metabarcoding applied to environmental impact assessment

PONE-D-26-15381R2

Dear Dr. Bouchet,

We’re pleased to inform you that your manuscript has been judged scientifically suitable for publication and will be formally accepted for publication once it meets all outstanding technical requirements.

Within one week, you’ll receive an e-mail detailing the required amendments. When these have been addressed, you’ll receive a formal acceptance letter and your manuscript will be scheduled for publication.

An invoice will be generated when your article is formally accepted. Please note, if your institution has a publishing partnership with PLOS and your article meets the relevant criteria, all or part of your publication costs will be covered. Please make sure your user information is up-to-date by logging into Editorial Manager at Editorial Manager® and clicking the ‘Update My Information' link at the top of the page. For questions related to billing, please contact billing support.

If your institution or institutions have a press office, please notify them about your upcoming paper to help maximize its impact. If they’ll be preparing press materials, please inform our press team as soon as possible -- no later than 48 hours after receiving the formal acceptance. Your manuscript will remain under strict press embargo until 2 pm Eastern Time on the date of publication. For more information, please contact onepress@plos.org.

Kind regards,

Claudio D'Iglio, Ph.D.

Academic Editor

PLOS One

Additional Editor Comments (optional):

Reviewers' comments:

# Editor report: Acceptance letter

**DOI:** 10.1371/journal.pone.0356357.r006

**Authors:** Claudio D'Iglio

PONE-D-26-15381R2

PLOS One

Dear Dr. Bouchet,

I'm pleased to inform you that your manuscript has been deemed suitable for publication in PLOS One. Congratulations! Your manuscript is now being handed over to our production team.

At this stage, our production department will prepare your paper for publication. This includes ensuring the following:

* All references, tables, and figures are properly cited

* All relevant supporting information is included in the manuscript submission,

* There are no issues that prevent the paper from being properly typeset

You will receive further instructions from the production team, including instructions on how to review your proof when it is ready. Please keep in mind that we are working through a large volume of accepted articles, so please give us a few days to review your paper and let you know the next and final steps.

Lastly, if your institution or institutions have a press office, please let them know about your upcoming paper now to help maximize its impact. If they'll be preparing press materials, please inform our press team within the next 48 hours. Your manuscript will remain under strict press embargo until 2 pm Eastern Time on the date of publication. For more information, please contact onepress@plos.org.

You will receive an invoice from PLOS for your publication fee after your manuscript has reached the completed accept phase. If you receive an email requesting payment before acceptance or for any other service, this may be a phishing scheme. Learn how to identify phishing emails and protect your accounts at https://explore.plos.org/phishing.

If we can help with anything else, please email us at customercare@plos.org.

Thank you for submitting your work to PLOS ONE and supporting open access.

Kind regards,

PLOS ONE Editorial Office Staff

on behalf of

Dr. Claudio D'Iglio

Academic Editor

PLOS One
